# RM1 Semiempirical Quantum Chemistry: Parameters for Trivalent Lanthanum, Cerium and Praseodymium

**DOI:** 10.1371/journal.pone.0124372

**Published:** 2015-07-01

**Authors:** José Diogo L. Dutra, Manoel A. M. Filho, Gerd B. Rocha, Alfredo M. Simas, Ricardo O. Freire

**Affiliations:** 1 Pople Computational Chemistry Laboratory, Departamento de Química, Universidade Federal de Sergipe, 49.100–000, São Cristóvão, SE, Brazil; 2 Departamento de Química, CCEN, Universidade Federal da Paraíba, 58.059–970, João Pessoa, PB, Brazil; 3 Departamento de Química Fundamental, Universidade Federal de Pernambuco, 50740–540, Recife, PE, Brazil; University of Calgary, CANADA

## Abstract

The RM1 model for the lanthanides is parameterized for complexes of the trications of lanthanum, cerium, and praseodymium. The semiempirical quantum chemical model core stands for the [Xe]4f^n^ electronic configuration, with n =0,1,2 for La(III), Ce(III), and Pr(III), respectively. In addition, the valence shell is described by three electrons in a set of 5d, 6s, and 6p orbitals. Results indicate that the present model is more accurate than the previous sparkle models, although these are still very good methods provided the ligands only possess oxygen or nitrogen atoms directly coordinated to the lanthanide ion. For all other different types of coordination, the present RM1 model for the lanthanides is much superior and must definitely be used. Overall, the accuracy of the model is of the order of 0.07Å for La(III) and Pr(III), and 0.08Å for Ce(III) for lanthanide-ligand atom distances which lie mostly around the 2.3Å to 2.6Å interval, implying an error around 3% only.

## Introduction

Lanthanum complexes find their usage as catalysts, for example, in the transesterification of triglycerides to monoesters [1], important in the making of biodiesel fuel, in the synthesis of novel antioxidants with high superoxide scavenging activity [2], in asymmetric epoxidation reactions [3], in P_4_ activation by lanthanum naphthalene complex [4], etc. Furthermore, lanthanum complexes may serve as extreme pressure lubrication additives in paraffin oil [5], they may display pH sensitivity [6], and are of interest to studies on chelator design [7] and polymer build up [8].

Cerium(III) complexes display low toxicity when compared to other lanthanide ions and are, for example, of interest in the design of new drugs targeting DNA [9]. They, of course, may also be used as catalysts, for example in the catalytic cleavage of phosphate esters, an important reaction which mimetizes the hydrolytic cleavage of DNA [10]. Also, due to the relative ease by which they can convert to Ce(IV), Ce(III) complexes may act as antioxidation agents, for example, as a hydroxyl radical quencher in fuel cell electrolyte membranes [11]. The structure of novel Ce(III) complexes are also thoroughly studied due to their several potential applications [12,13].

The 4f^2^ electronic configuration of Pr(III) gives rise to a series of electronic states, and therefore luminescence of Pr(III) covers a wide range of wavelengths, ranging from the ultraviolet to the near infrared. However, Pr(III) luminescence is rarely observed in the visible region[14]–the most important use of Pr(III) complexes in luminescent and electroluminescent devices being as near infrared emitters [15]. Biological activities of praseodymium complexes have also been observed due to their substantial affinities for many biomolecules, as are the cases of DNA-binding [16] and their presence in Schiff-base complexes [17]. Further, the large variety of architectures of Pr(III) complexes have been receiving continued interest, as novel complexes are being reported [18], including a complex of Pr(III) with organofluorotitanate ligands with coordination number 12 [19]. Furthermore, larger clusters are being prepared, as is the case of the tetramer [Pr_4_Cl_10_(OH)_2_(thiazole)_8_(H_2_O)_2_], the first thiazole complex of a lanthanide ion reported [20], and of the three-dimensional 5-aminoisophtalate Pr(III) polymeric complex which presents good gas storage capabilities [21].

Therefore, the theoretical modeling of complexes of La(III), Ce(III), and Pr(III) is an open area of research, important for the selection of ligands, of counter ions, of specific coordination geometries, of metal to ligand ratios, of polymer framework topologies, of thermal and photostability, for the fine tuning of optical and magnetic properties, and so on.

Previously, our research group introduced the Sparkle Model [22,23], originally to allow the calculation of Eu(III) complexes within the semiempirical AM1 model [24], together with a prescription to compute the UV-Visible electronic spectra of the complexes using another semiempirical model, INDO/S [25–28]. The model was then successfully applied to the design of luminescent Eu(III) complexes [29–32] and proven useful for the prediction of ligand field parameters [33]. In 2004, we introduced Gaussian functions in the core-core repulsion of our sparkle model in order to make it consistent with AM1, something that greatly improved the accuracy of the model [34]. Thus, in 2005 we introduced the new model, we called Sparkle/AM1 for all trivalent lanthanide ions [35]. Since then, we have further introduced the Sparkle Model for La (III), Ce (III), and Pr (III) for PM3 [36–38], for PM6 [39], for PM7 [40], and RM1 [41].

The sparkle model proved very accurate for more ionic bonds of the hard-hard type, such as the cases of directly coordinated oxygen and nitrogen atoms. However, the sparkle model fails when other types of atoms are directly coordinated to the lanthanide ion, as is the cases of carbon, sulfur, and the heavier halogens.

In order to be able to address all types of bonds between the central trivalent lanthanide ion and its ligands, we introduced in 2013 a new and more perfected model, we called the RM1 model for the lanthanides, and presented parameters for Eu(III), Gd(III), Tb(III) [42], Dy(III), Ho(III), and Er(III) [43]. In this model, we considered the lanthanide ion to be a regular neutral atom within RM1 [44] where the semiempirical core represents the electronic configuration [Xe]4f^n^, with n varying from 0 for La (III) to 14 for Lu (III), and we add three electrons to a valence shell comprised of the semiempirical atomic orbitals 5d, 6s, and 6p. This new RM1 model for the lanthanides proved to be very general and capable of much more accurately describing the multitude of different bonds that show up in lanthanide chemistry.

In the present article, we further extend the RM1 model for the lanthanides to complexes of La(III), Ce(III), and Pr(III).

## Method

As indicated before, the RM1 model for the lanthanides assumes that the electronic configuration [Xe]4f^n^ with n = 0,1,2, for La(III), Ce(III) and Pr(III), respectively, can be correctly described by the semiempirical core of charge +3. In addition, the model attaches a set of semiempirical 5d, 6s, and 6p orbitals to describe the valence shell, which always contains 3 electrons for all lanthanide trications. As a result, 22 parameters need to be optimized for each of the lanthanides.

A usual and recurrent criticism of semiempirical models is that they tend to perform well for systems for which they were parameterized, and tend to perform poorly or even badly for other systems. In order to minimize this, we created, in our research group, a method of parameterization which seeks to obtain much more robust models [34,35,45]. We start by collecting all existing complexes of the lanthanide ion of interest that can be found in the Cambridge Structural Database (CSD) [46–48]. In order to guarantee quality in our parameters, we restrict ourselves to collect only complexes of high crystallographic quality (R < 0.05). Of course, we understand that, due to their unique characteristics, each lanthanide metal has a particular palette of applications, each requiring their own specific type of ligands. Therefore, we assume that the more useful complexes will be naturally more numerous in the universe of high quality structures of the CSD database for each particular metal. Having collected that, we note that there is no point in parameterizing the model for all existing high quality CSD complexes simultaneously because, there could be many repeating ligands, which would be overrepresented in the parameterization set and which could cause an imbalance in the parameters. Therefore, at this point, we need to select sub-sets of complexes to serve as parameterization sets. In addition, this selection must take into account the relative difficulty of predicting, from quantum chemical calculations, the geometries of the complexes. For the purpose of this selection, we assume that a good measure of this difficulty is the difference between the crystallographic geometries and geometries obtained by our previous model Sparkle/AM1 [37,38,49]. Thus, for each complex i, we define the following measure R_i_:

$$R_{i}=\sum_{j}\sum_{k}\frac{1}{\sigma_{j}^{dist}}\left|d_{i,j,k}^{CSD}-d_{i,j,k}^{Calc}\right|+\sum_{l}\frac{1}{\sigma^{angle}}\left|\theta_{i,\text{l}}^{CSD}-\theta_{i,\text{l}}^{Calc}\right|$$ (1)

where d refers to distances, and θ to angles; CSD refers to data obtained from the CSD, and Calc refers to data obtained from our previous model calculation (Sparkle/RM1); j runs over all types of bonds, e.g. Ln-O, Ln-N, Ln-C, etc, and k runs over all bonds of the j type; $\sigma_{j}^{dist}$ is the standard deviation of all differences between CSD and Sparkle/AM1 (calc) for all bonds of the j type; l runs over all angles; and $\sigma^{angle}$ is the standard deviation of all angle differences between CSD and Sparkle/AM1 (calc). The set of measures R_i_ was then used as input for a divisive hierarchical clustering analysis, DIANA [50], from which we selected two parameterization sets from the universes of complexes for each lanthanide metal: one we call the small set, with only 15 complexes for La(III), 8 complexes for Ce(III), and 7 complexes for Pr(III); and another one we call the large parameterization set, with 38 complexes for La(III), 18 for Ce(III), and 16 for Pr(III). The next step, is the optimization of the model where, by means of a combination of a few non-linear optimization techniques, we seek to minimize a response function, which is the sum of all R_i_s of Eq (1), with the difference that calc will now refer to the particular distance or angle calculated by means of the intermediary set of parameters of the optimization procedure. When the nonlinear optimization process converges for the small set of complexes, we start it all over again with the large set. Finally, we declare the process of nonlinear optimization to be finished when it converges for the large set.

Assessments of the accuracy of the model can be made via the unsigned mean error, UME_i_, defined for each complex i as

$$UME_{i}=\frac{1}{n}\sum_{j=1}^{n}\left|R_{i,j}^{CSD}-R_{i,j}^{Calc}\right|$$ (2)

where CSD and Calc are as in Eq (1), and the summation runs over all the n bonds being considered. As before, we use two different measures: UME_(Ln-L)i_ and UME_i_. The first contains all j distances between the lanthanide ion and its directly coordinated atoms. The second, includes, in addition, all distances between all directly coordinated atoms and indirectly also reflects a measure of the accuracy of the predicted angles within the coordination polyhedron.

The next step in verifying the robustness of the parameterization is to determine if the distribution of unsigned mean deviations between the predicted and crystallographic geometries can be adequately described by a gamma distribution function. That can be ascertained, by means of the one-sample nonparametric Kolmogorov-Smirnoff test whose p-value must be larger than 0.05, indicating that usage of the mean and variance of the gamma distribution fit as accuracy measures of the models are statistically justified within a 95% level of confidence.

Table 1 presents the three sets of 22 RM1 parameters found for Ln(III), Ce(III), and Pr(III). And Tables 2 and 3 present the mean and variance of the gamma distribution fits for the both types of unsigned mean errors for the universe of complexes, together with the p-value which is larger than 0.05 for all cases. All that indicates that the RM1 models here advanced for La(III), Ce(III), and Pr(III) are capable of predicting the geometries of the corresponding complexes in a reliable manner, and that the eventual deviations from the experiment behave as random around the correct values.

**Table 1:** Parameters* for the RM1 model for the trications of La, Ce and Pr.

RM1			
	La^3+^	Ce^3+^	Pr^3+^
*U*_*ss*_	-14.68043413	-14.71938888	-14.52408063
*U*_*pp*_	-6.73473860	-7.68942949	-7.05682683
*U*_*dd*_	-20.48996706	-20.45157682	-20.68932756
*ζ*_*s*_	1.27267748	1.28102807	1.53803892
*ζ*_*p*_	1.42327584	1.42536636	1.58164715
*ζ*_*d*_	1.41036886	1.41286566	1.37490374
*β*_*s*_	-7.66955512	-7.66878654	-7.94799309
*β*_*p*_	0.47769647	0.44444183	0.85381597
*β*_*d*_	-3.71147661	-3.74493844	-3.83029281
*F0SD*	7.72081332	7.71512874	7.61830081
*G2SD*	3.91674532	3.91829281	3.96586318
*POC*	1.87517566	1.87508416	1.84741576
*α*	1.28404676	1.28623319	1.28060282
*ZSN*	0.78452960	0.81941318	0.78194425
*ZPN*	1.50661174	1.43034873	1.29298193
*ZDN*	1.17206190	1.19321984	0.98960461
*a*_*11*_	0.62855906	0.66196096	0.45337932
*b*_*21*_	7.86084906	7.89025802	7.82319885
*c*_*31*_	1.30447476	1.26282727	1.56516739
*a*_*12*_	0.08164180	0.07599228	0.01039415
*b*_*22*_	10.34685773	10.31610935	10.28836535
*c*_*32*_	3.24704021	3.24781703	3.26870321

*Parameters are *s*, *p*, and *d* atomic orbital one-electron one-center integrals *U*_*ss*_, *U*_*pp*_ and *U*_*dd*_; the *s*, *p*, and *d* Slater atomic orbital exponents *ξ*_*s*_, *ξ*_*p*_, and *ξ*_*d*_; the *s*, *p*, and *d* atomic orbital one-electron two-center resonance integral terms *β*_*s*_, *β*_*p*_, and *β*_*d*_; the core-core repulsion term *α*; the two-electron integrals F^0^_SD_, G^2^_SD_; and the additive term ρ_core_ needed to evaluate core-electron and core-core nuclear interactions; the second set of exponents to compute the one-center integrals *ξ*_*s*_ *’*, *ξ*_*p*_ *’*, and *ξ*_*d*_ *’*; and the six parameters for the two Gaussian functions: height, a_i_; inverse broadness, b_i_; and displacement, c_i_; as in $G(R)=\sum_{i=1}^{2}a_{i}e^{\left[b_{i}{\left(R-c_{i}\right)}^{2}\right]}$ where R, is the interatomic distance between the lanthanide and the other atom.

**Table 2:** Means and variances of the *γ* distribution fits for the UME_(Ln-L)_s computed for the *N* complexes for each lanthanide trication.

UME_(Ln-L)_s				
lanthanide ion	N	mean (Å)	variance (Å^2^)	p-value
La^3+^	84	0.0710	0.0032	0.1418
Ce^3+^	57	0.0805	0.0040	0.9630
Pr^3+^	65	0.0685	0.0037	0.6141

**Table 3:** Means and Variances of the *γ* distribution fits for the UMEs computed for the *N* complexes for each lanthanide trication.

UMEs				
lanthanide ion	N	mean (Å)	variance (Å^2^)	p-value
La^3+^	84	0.1524	0.0276	0.7502
Ce^3+^	57	0.1621	0.0373	0.2089
Pr^3+^	65	0.1790	0.0469	0.5420

## Results and Discussion

Table 4 presents unsigned mean errors for each of the specific types of distances between the lanthanide ion and its directly coordinated atoms found in the universe of complexes for La(III), both for the present RM1 model for the lanthanides and for each of the previous sparkle models. In order to facilitate interpretation of the table, the smallest error in each line is being bolded. Clearly, for dinuclear complexes, the La-La bond is more accurately predicted by Sparkle/PM3. However, its error is relatively close to the RM1 error. The same happens for La-O bonds, where Sparkle/PM3 is again the best model. However, its unsigned mean error of 0.0610Å is too close to the RM1 error of 0.0698Å. However, for all other distances, RM1 presents the smallest errors while the previous Sparkle models sometimes display huge errors as is the case of La-S bonds when the average errors of the Sparkle models is 0.4345Å, a value more than 6 times larger than the RM1 error of 0.0680Å. In Table 4, La-L refers to the unsigned mean error of all distances of all types between the central lanthanum ion and its directly coordinated other atoms, whereas L-L includes all interatomic distances between all directly coordinated atoms, and is, indirectly, a measure of the angles within the coordinated polyhedron. Clearly, RM1, with its unsigned mean error of 0.1704Å is 52% smaller than the average of the previous sparkle models, a situation similar to what happens to the next unsigned mean error, which includes all 5315 types of distances for all lanthanum complexes considered: La-L, La-La, and L,L’, when RM1 displays an error which is 56% smaller than the average error of all previous sparkle models.

**Table 4:** Sparkle/AM1, Sparkle/PM3, Sparkle/PM6, Sparkle/PM7, Sparkle/RM1 and RM1 unsigned mean errors for lanthanum(III) complexes.

Type of distances	unsigned mean errors for specific types of distances (Å)						
	N	RM1	Sparkle				
			AM1	PM3	PM6	PM7	RM1
La—La	13	0.1807	0.2056	**0.1404**	0.2153	0.1900	0.2963
La—O	580	0.0698	0.0853	**0.0610**	0.0767	0.0962	0.1252
La—N	205	**0.0487**	0.0541	0.0873	0.0685	0.2437	0.0673
La—C	119	**0.0980**	0.2193	0.2681	0.2120	0.2771	0.2114
La—S	15	**0.0680**	0.4018	0.4302	0.3847	0.5240	0.4316
La—Cl	26	**0.0946**	0.3033	0.3441	0.2836	0.3693	0.3517
La—Br	4	**0.0957**	0.3974	0.4282	0.4009	1.7007	0.4742
La—L	962	**0.0710**	0.1092	0.1087	0.1044	0.1697	0.2144
L—L	4353	**0.1704**	0.2334	0.2300	0.2521	0.2857	0.2941
La-L, La—La and L-L’	5315	**0.1524**	0.2110	0.2081	0.2255	0.2648	0.2809

Tables 5 and 6 show equivalent results for Ce(III) and for Pr(III) complexes. For Ce(III) complexes, RM1 is more accurate when compared to the other sparkle models with respect to all measures except for Ce-O distances. Likewise, for Pr(III) complexes, RM1 is more accurate than the other sparkle models except for Pr-O and Pr-N distances. Even in these cases, the accuracy of RM1 is close to the best accuracy available from the other sparkle models. A noteworthy case, are the distances of the three lanthanide ions and bromine, where Sparkle/PM7 displays enormous unsigned mean errors, larger than 1Å, suggesting there might be perhaps some difficulties with the parameterization of bromine in PM7.

**Table 5:** Sparkle/AM1, Sparkle/PM3, Sparkle/PM6, Sparkle/PM7, Sparkle/RM1 and RM1 unsigned mean errors for cerium(III) complexes.

Type of distances	unsigned mean errors for specific types of distances (Å)						
	N	RM1	Sparkle				
			AM1	PM3	PM6	PM7	RM1
Ce—Ce	5	**0.1781**	0.2003	0.2022	0.2811	0.1271	0.1484
Ce—O	283	0.0900	0.0787	**0.0746**	0.1662	0.1564	0.0800
Ce—N	111	**0.0622**	0.0836	0.0708	0.0580	0.0636	0.0753
Ce—C	185	**0.0800**	0.2307	0.2524	0.1477	0.3390	0.2602
Ce—S	11	**0.0434**	0.4792	0.4607	0.3801	0.3074	0.4380
Ce—Cl	17	**0.0609**	0.2647	0.2882	0.1999	0.1604	0.2969
Ce—Br	9	**0.0378**	0.3757	0.3823	0.3159	1.4764	0.3934
Ce—L	622	**0.0805**	0.1406	0.1436	0.1641	0.2054	0.1434
L—L	3030	**0.1789**	0.2421	0.2507	0.2504	0.3078	0.2597
Ce—L, Ce—Ce and L—L’	3652	**0.1621**	0.2249	0.2326	0.2373	0.2903	0.2399

**Table 6:** Sparkle/AM1, Sparkle/PM3, Sparkle/PM6, Sparkle/PM7, Sparkle/RM1 and RM1 unsigned mean errors for praseodymium(III) complexes.

Type of distances	unsigned mean errors for specific types of distances (Å)						
	N	RM1	Sparkle				
			AM1	PM3	PM6	PM7	RM1
Pr—Pr	11	**0.1770**	0.2278	0.1998	0.2232	0.2673	0.2229
Pr—O	421	0.0736	0.0786	0.0841	0.0732	0.1044	**0.0723**
Pr—N	119	0.0626	0.1183	0.1014	0.0714	**0.0534**	0.1223
Pr—C	143	**0.0552**	0.2446	0.2536	0.2322	0.3591	0.2211
Pr—S	12	**0.0787**	0.3817	0.3870	0.4304	0.5795	0.3898
Pr—Cl	39	**0.0526**	0.2549	0.2739	0.2899	0.2975	0.3700
Pr—Br	6	**0.0251**	0.3460	0.3509	0.3814	1.2262	0.4627
Pr—L	751	**0.0685**	0.1348	0.1376	0.1248	0.1738	0.1318
L—L	3462	**0.2029**	0.2621	0.2401	0.2628	0.2893	0.2566
Pr—L, Pr—Pr and L—L’	4213	**0.1790**	0.2394	0.2218	0.2382	0.2687	0.2345

**Fig 1:**
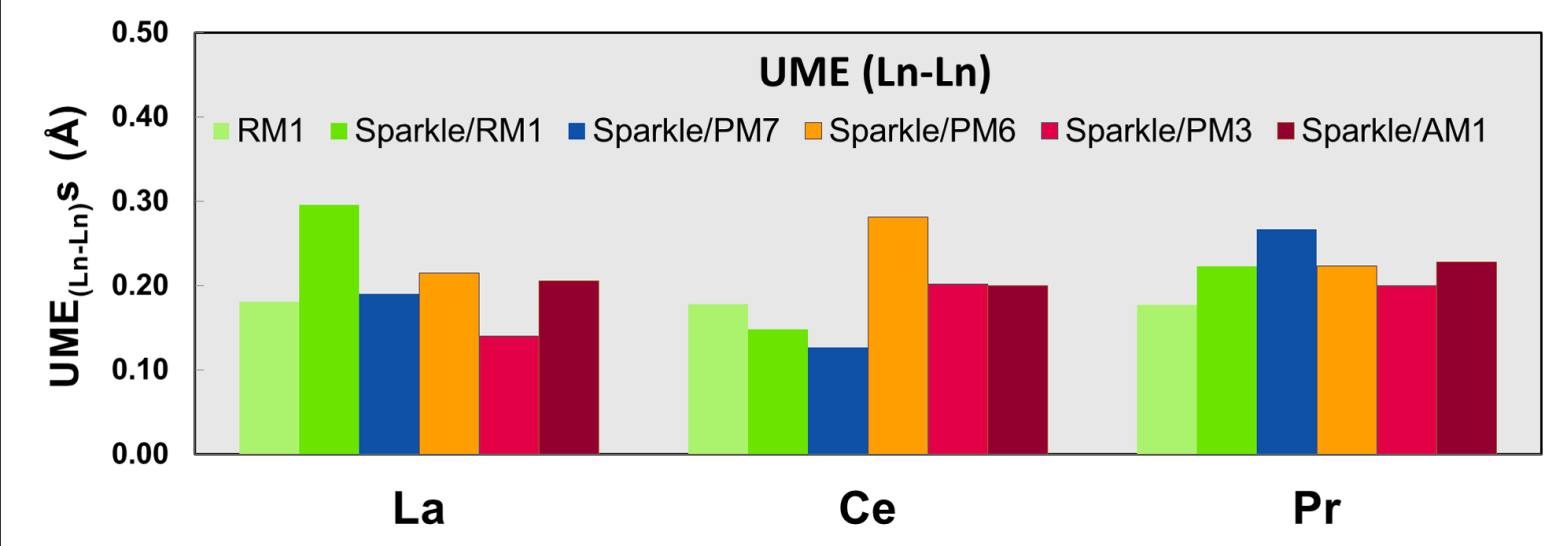
UME_(Ln-Ln)_s obtained using the RM1 model for the lanthanides and all five versions of the Sparkle Model: Sparkle/RM1, Sparkle/PM7, Sparkle/PM6, Sparkle/PM3 and Sparkle/AM1 for all complexes of the universe set for each of the lanthanide trications: La(III), Ce(III) and Pr(III). The UMEs are calculated as the absolute value of the difference between the experimental and calculated Ln-Ln interatomic distances, summed up for all complexes, for each of the lanthanides.

**Fig 2:**
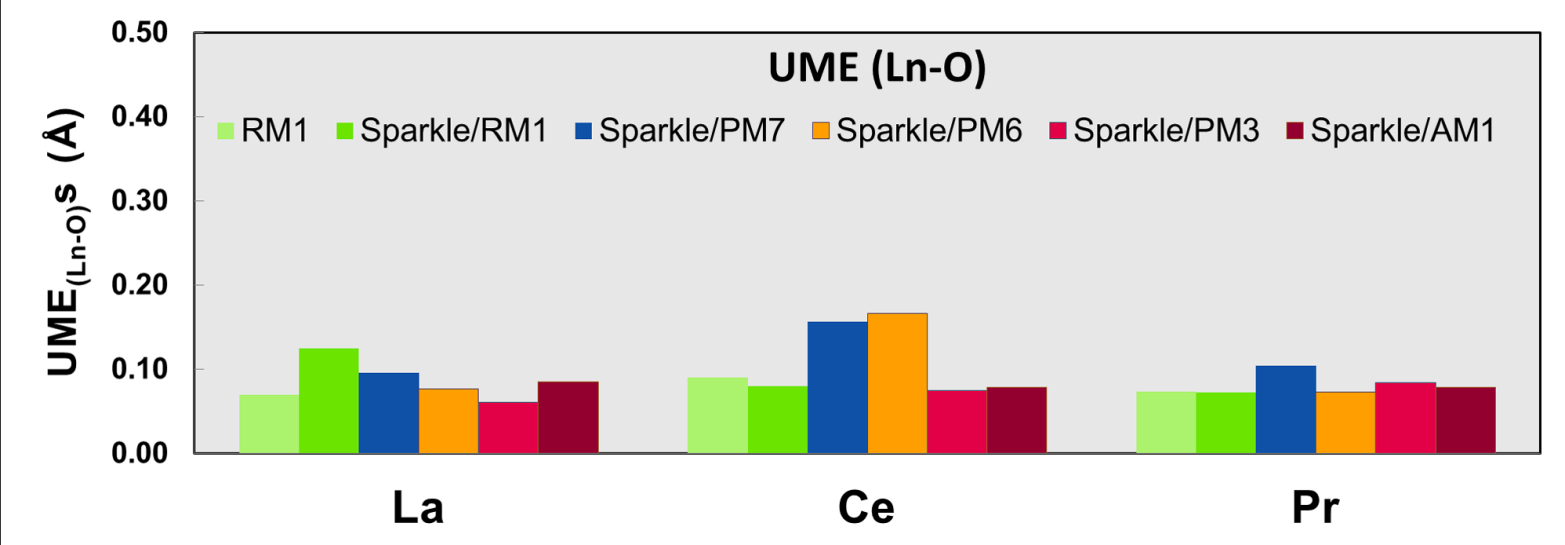
UME_(Ln-O)_s obtained using the RM1 model for the lanthanides and all five versions of the Sparkle Model: Sparkle/RM1, Sparkle/PM7, Sparkle/PM6, Sparkle/PM3 and Sparkle/AM1 for all complexes of the universe set for each of the lanthanide trications: La(III), Ce(III) and Pr(III). The UMEs are calculated as the absolute value of the difference between the experimental and calculated Ln-O interatomic distances, summed up for all complexes, for each of the lanthanides.

**Fig 3:**
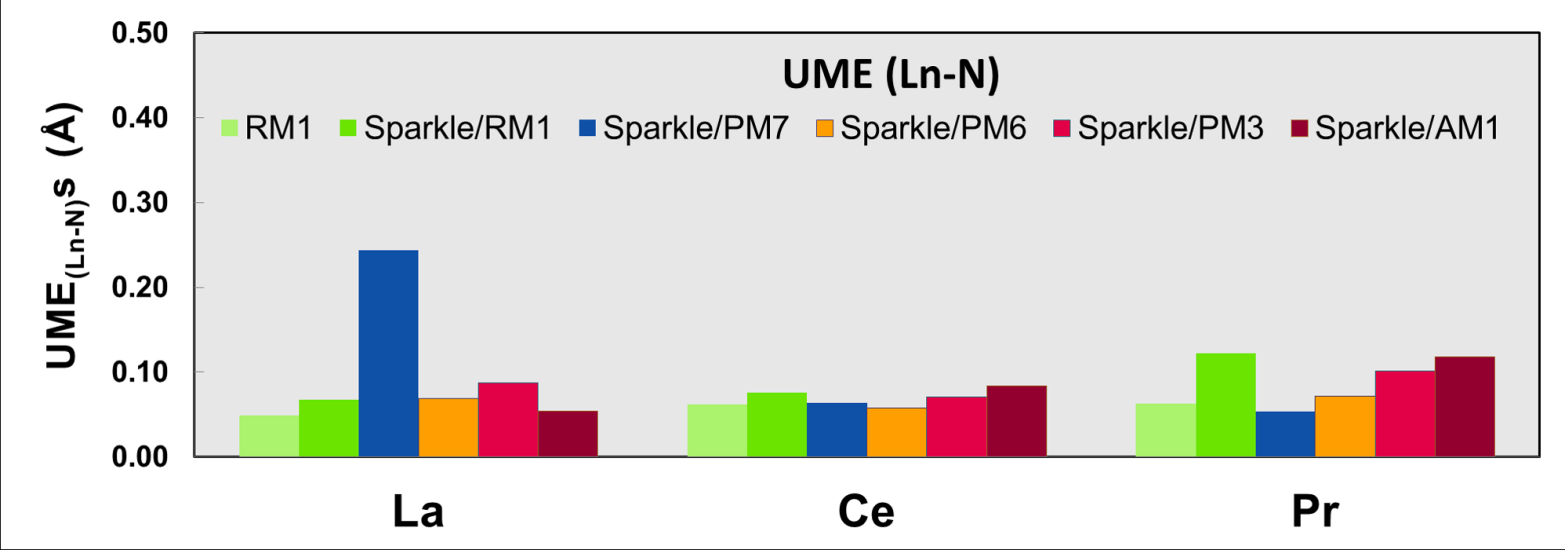
UME_(Ln-N)_s obtained using the RM1 model for the lanthanides and all five versions of the Sparkle Model: Sparkle/RM1, Sparkle/PM7, Sparkle/PM6, Sparkle/PM3 and Sparkle/AM1 for all complexes of the universe set for each of the lanthanide trications: La(III), Ce(III) and Pr(III). The UMEs are calculated as the absolute value of the difference between the experimental and calculated Ln-N interatomic distances, summed up for all complexes, for each of the lanthanides.

**Fig 4:**
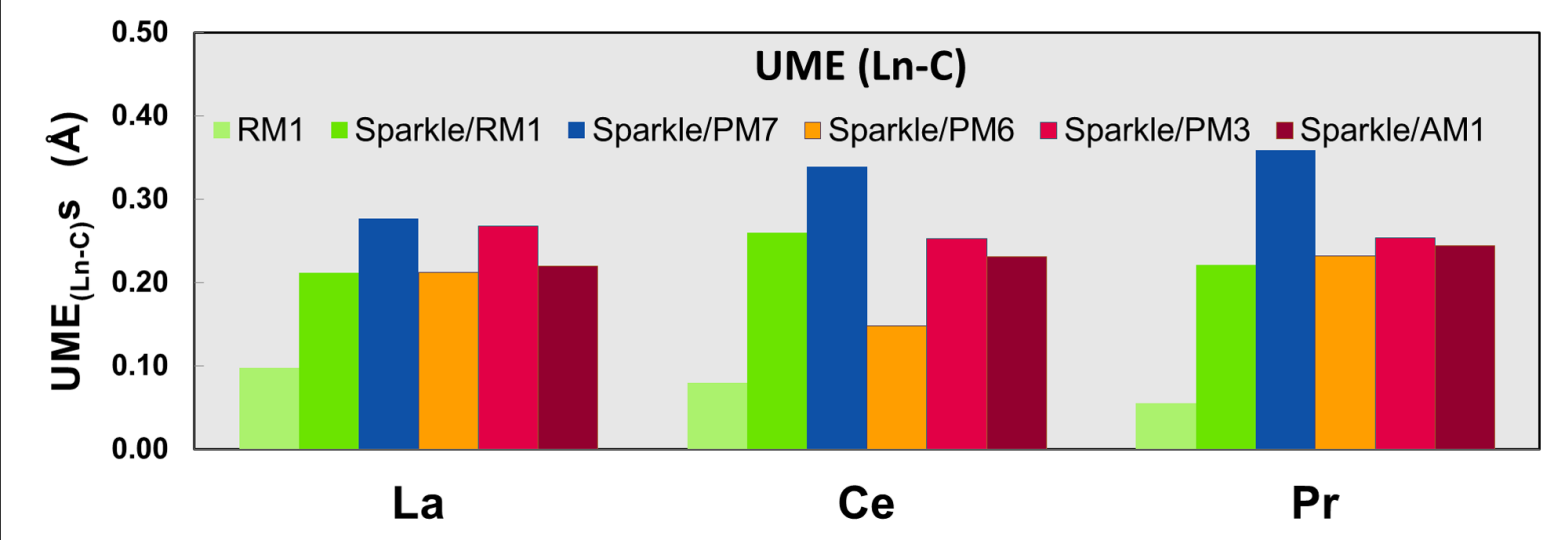
UME_(Ln-C)_ obtained using the RM1 model for the lanthanides and all five versions of the Sparkle Model: Sparkle/RM1, Sparkle/PM7, Sparkle/PM6, Sparkle/PM3 and Sparkle/AM1 for all complexes of the universe set for each of the lanthanide trications: La(III), Ce(III) and Pr(III). The UMEs are calculated as the absolute value of the difference between the experimental and calculated Ln-C interatomic distances, summed up for all complexes, for each of the lanthanides.

**Fig 5:**
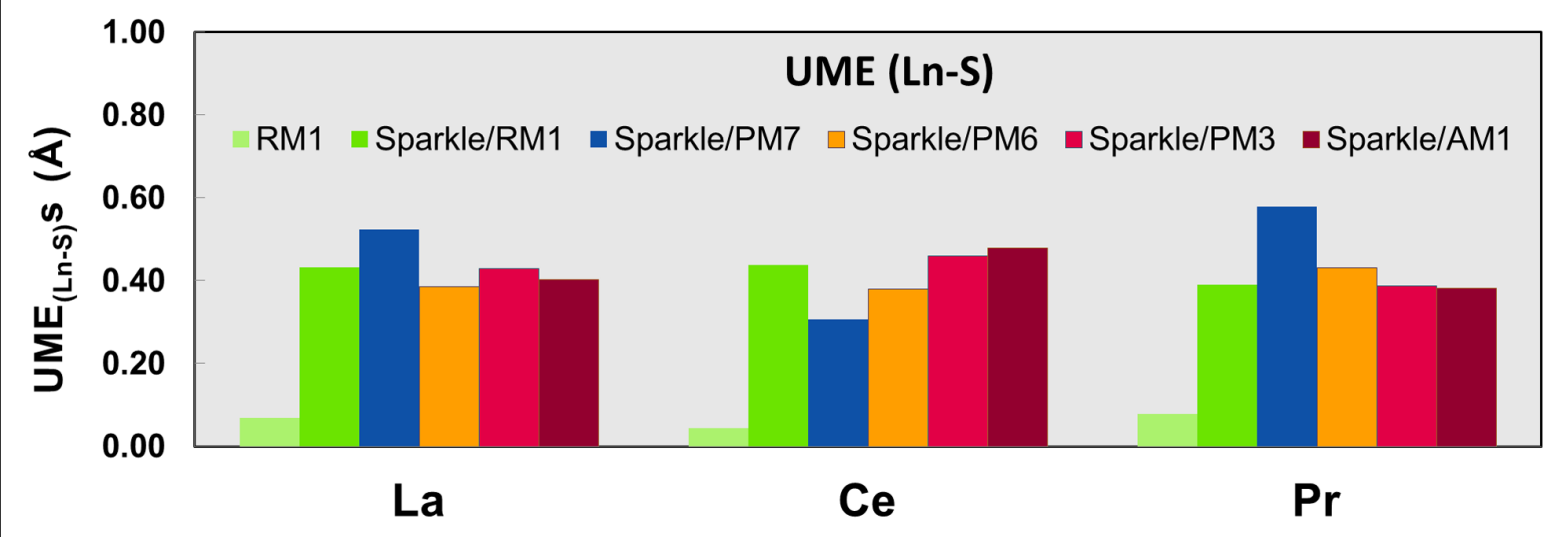
UME_(Ln-S)_ obtained using the RM1 model for the lanthanides and all five versions of the Sparkle Model: Sparkle/RM1, Sparkle/PM7, Sparkle/PM6, Sparkle/PM3 and Sparkle/AM1 for all complexes of the universe set for each of the lanthanide trications: La(III), Ce(III) and Pr(III). The UMEs are calculated as the absolute value of the difference between the experimental and calculated Ln-S interatomic distances, summed up for all complexes, for each of the lanthanides. There are no Ho-S distances in the universe of Ho(III) complexes considered.

**Fig 6:**
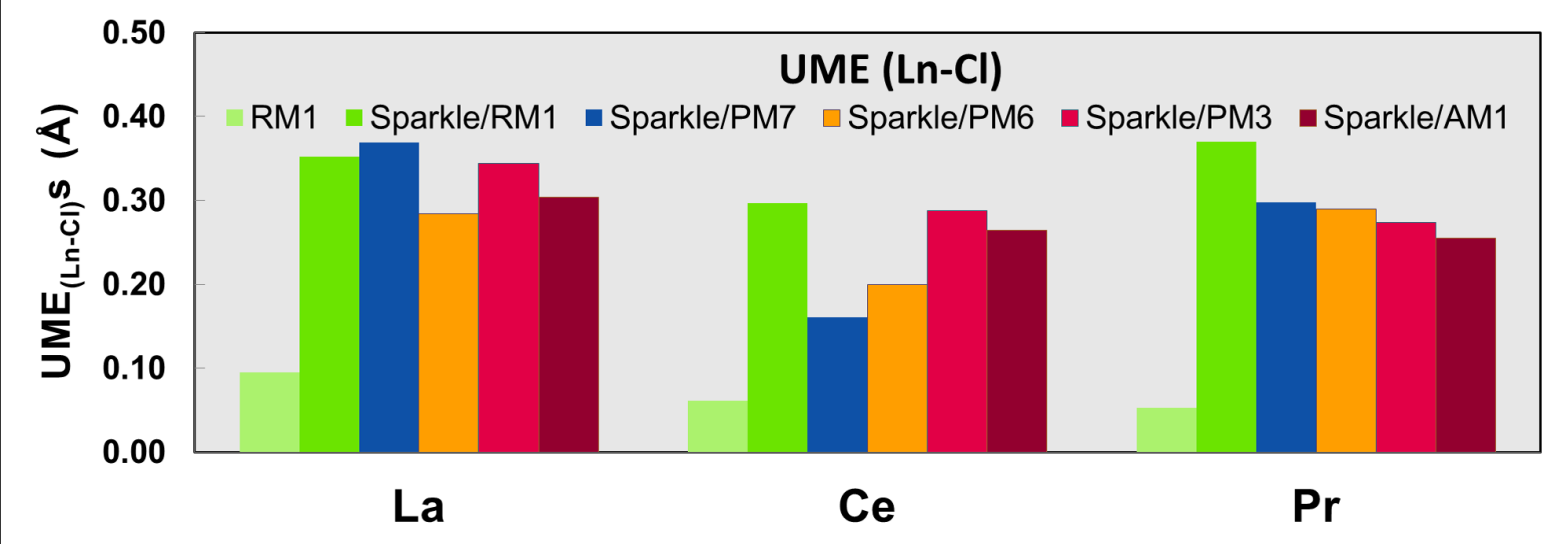
UME_(Ln-Cl)_s obtained using the RM1 model for the lanthanides and all five versions of the Sparkle Model: Sparkle/RM1, Sparkle/PM7, Sparkle/PM6, Sparkle/PM3 and Sparkle/AM1 for all complexes of the universe set for each of the lanthanide trications: La(III), Ce(III) and Pr(III). The UMEs are calculated as the absolute value of the difference between the experimental and calculated Ln-Cl interatomic distances, summed up for all complexes, for each of the lanthanides.

**Fig 7:**
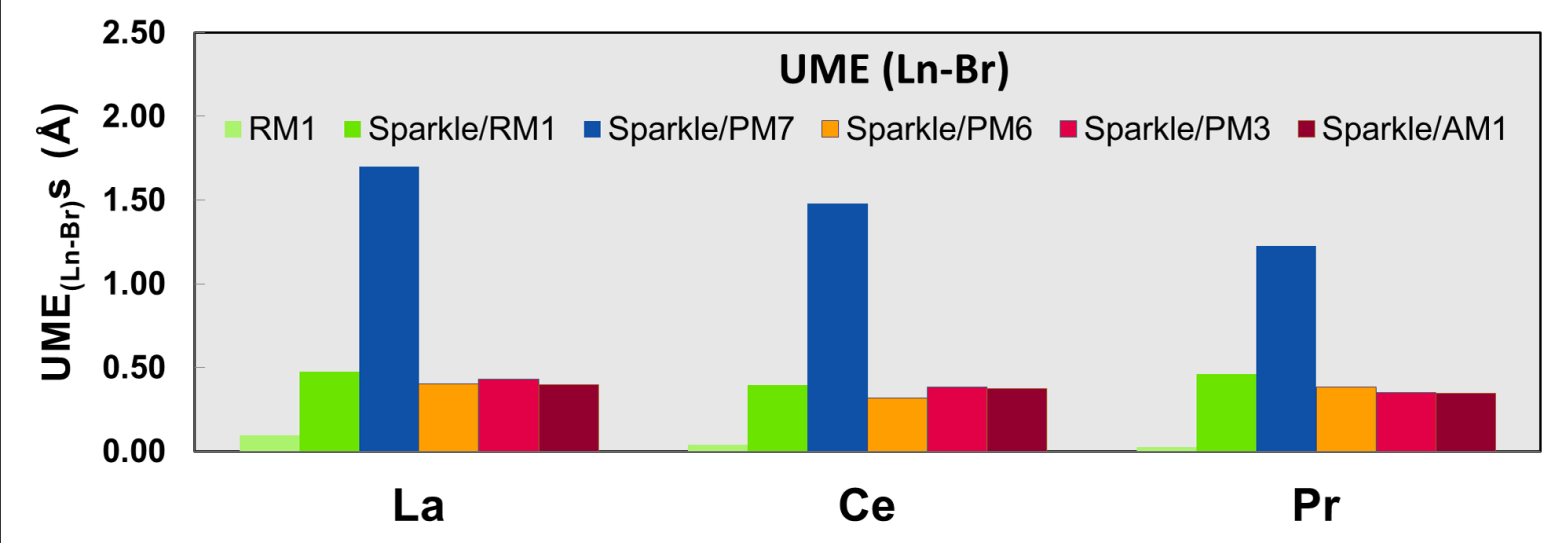
UME_(Ln-Br)_s obtained using the RM1 model for the lanthanides and all five versions of the Sparkle Model: Sparkle/RM1, Sparkle/PM7, Sparkle/PM6, Sparkle/PM3 and Sparkle/AM1 for all complexes of the universe set for each of the lanthanide trications: La(III), Ce(III) and Pr(III). The UMEs are calculated as the absolute value of the difference between the experimental and calculated Ln-Br interatomic distances, summed up for all complexes, for each of the lanthanides.

**Fig 8:**
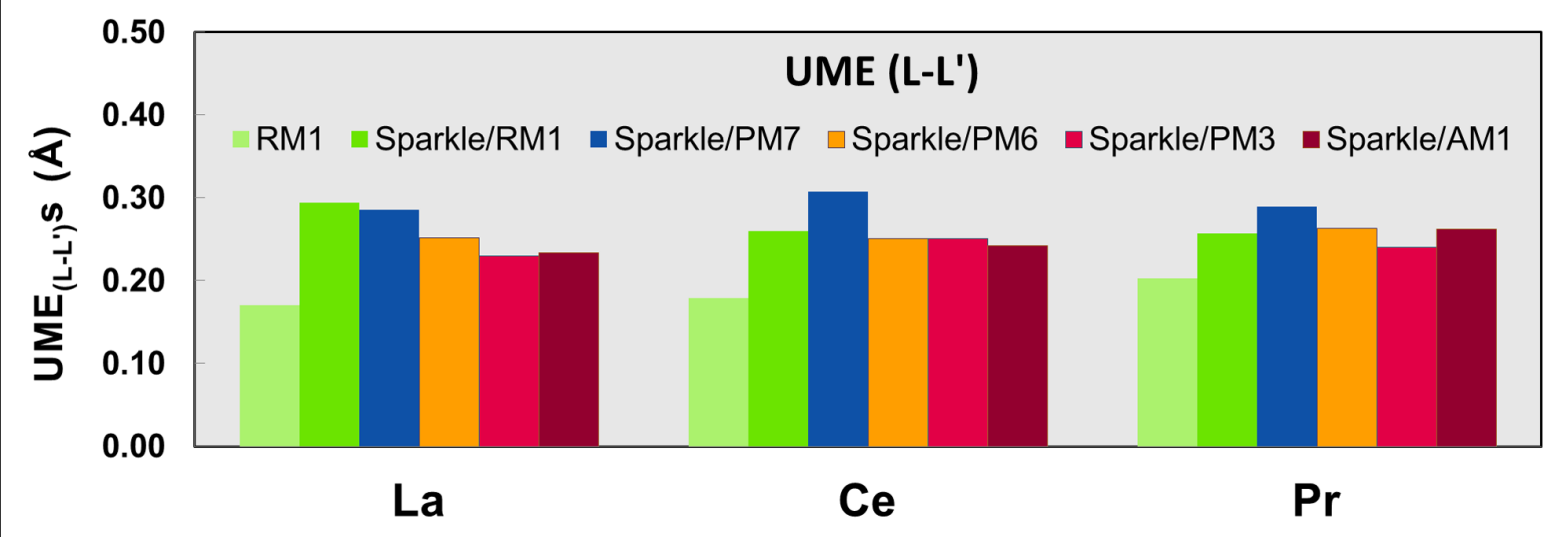
UME_(L-L’)_s obtained using the RM1 model for the lanthanides and all five versions of the Sparkle Model: Sparkle/RM1, Sparkle/PM7, Sparkle/PM6, Sparkle/PM3 and Sparkle/AM1 for all complexes of the universe set for each of the lanthanide trications: La(III), Ce(III) and Pr(III). The UMEs are calculated as the absolute value of the difference between the experimental and calculated interatomic distances between the coordinated atoms, L-L’, summed up for all complexes, for each of the lanthanides.

**Fig 9:**
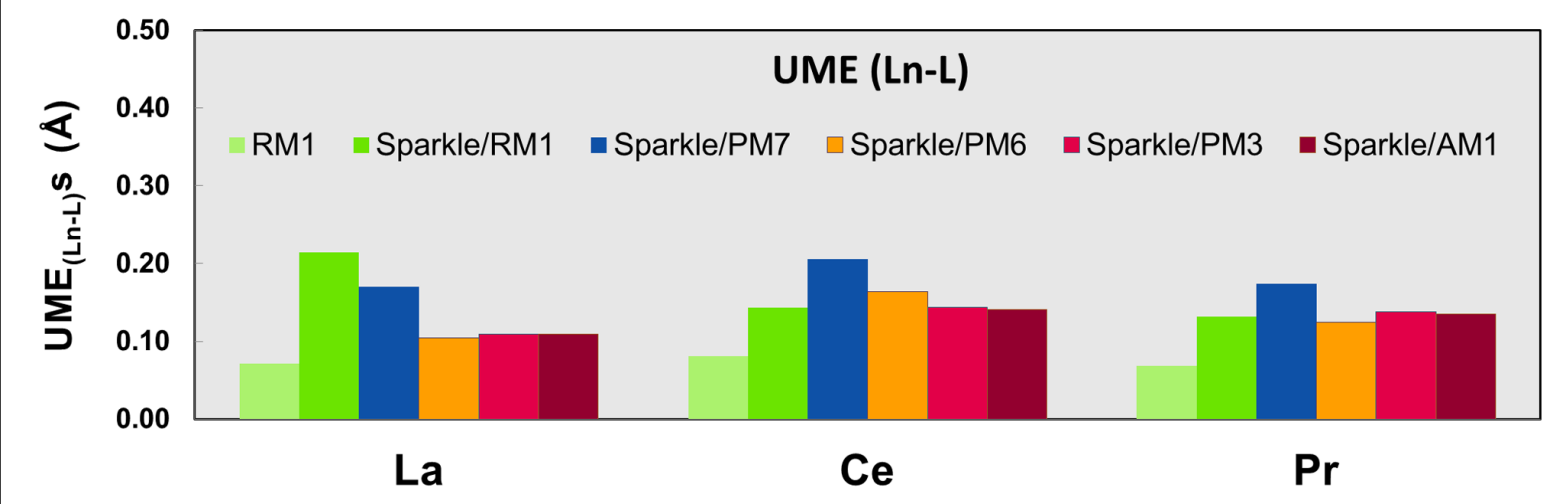
UME_(Ln-L’)_s obtained using the RM1 model for the lanthanides and all five versions of the Sparkle Model: Sparkle/RM1, Sparkle/PM7, Sparkle/PM6, Sparkle/PM3 and Sparkle/AM1 for all complexes of the universe set for each of the lanthanide trications: La(III), Ce(III) and Pr(III).

**Fig 10:**
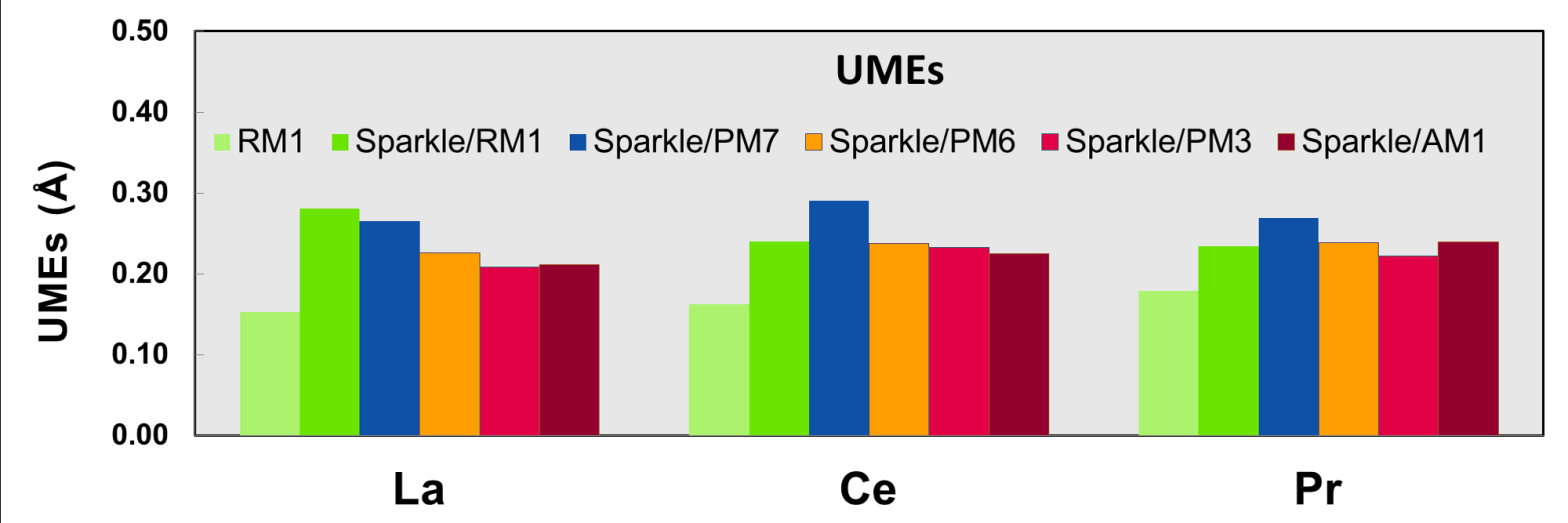
UMEs obtained using the RM1 model for the lanthanides and all five versions of the Sparkle Model: Sparkle/RM1, Sparkle/PM7, Sparkle/PM6, Sparkle/PM3 and Sparkle/AM1 for all complexes of the universe set for each of the lanthanide trications: La(III), Ce(III) and Pr(III).

Figs 1–10 present the data in Tables 3–5 in pictorial form in order to provide the user with an instant comprehension of the relative accuracies of the present RM1 model for the lanthanides (light green bar) and the previous sparkle models. Note that the Sparkle/RM1 green bar lies next, followed by the blue bar of Sparkle/PM7. Thus, the situation highlighted above on the inadequacy of Sparkle/PM7 for any of the parameterized lanthanide ions when directly bonded to bromine can be immediately detected in Fig 7. On the bright side, the good accuracy of all previous sparkle models for directly coordinated oxygen and nitrogen bonds is very clearly manifested (Figs 2 and 3).

Finally, the raw data used to arrive at the values presented in Tables 4–6, can be found in Tables 7, 8, and 9, which show individual unsigned mean errors for each of the complexes considered, and identifies by a underlined and bolded codes, the complexes used in the small and large parameterization sets.

**Table 7:** Unsigned mean errors, UME_(Eu-L)_s and UMEs, for RM1 model for lanthanides, as compared to the respective experimental crystallographic values, obtained from the Cambridge Structural Database, for each of the 84 lanthanum(III) complexes.

Structure	RM1		Structure	RM1	
	UME_(La-L)_s (Å)	UME (Å)		UME_(La-L)_s (Å)	UME (Å)
**ABXALA**	0.0684	0.1722	NERQUW	0.1504	0.2095
ALANIC	0.0676	0.1159	**NOHNIH**	0.0479	0.1353
APBNLA	0.0513	0.1067	OFEGIP	0.0304	0.1433
**AXOMOP**	0.1060	0.1183	**PAFNEP**	0.0394	0.1035
**AYULUB**	0.1187	0.1565	**PIBGOW**	0.0679	0.2653
BEQPOC	0.0593	0.2330	PIRSEO	0.0879	0.1403
**BIZTIN**	0.0614	0.2370	POHDUL	0.0640	0.2192
**BOKZUX**	0.0536	0.1135	**PUHYAS**	0.0488	0.1787
**BUVVIX01**	0.0559	0.1049	PUWZIQ	0.0615	0.1252
CABLAS01	0.0603	0.1862	PUZHOH	0.0406	0.1630
CEFQOT	0.0619	0.0820	QAKWEE	0.0499	0.1854
**CESRUO**	0.0983	0.1775	QAPXAG	0.0590	0.1403
**COTDOF**	0.1244	0.1221	QUBWIT	0.0432	0.0859
DUBWEC	0.0401	0.1174	**RIWQOE**	0.0245	0.3574
DUCBOS	0.0688	0.1450	**SILWEQ**	0.0486	0.3046
EBEGOH	0.0530	0.1483	SIXBIK	0.0515	0.1749
**EPAILA**	0.0398	0.1493	SUXLIG	0.0435	0.0876
**EZIPUY**	0.0094	0.1105	SUZXIU	0.0672	0.1007
FABPUT	0.0875	0.1234	**TEPSOW**	0.0758	0.2000
FICJEG	0.0410	0.2938	TEQBIA	0.0559	0.2320
**FIVCIW**	0.0262	0.0392	TEQBOG	0.0556	0.1459
**FURLOT**	0.0976	0.1405	TUPWEG	0.0552	0.0808
GIMMIY	0.0487	0.2507	VUBLIN	0.1069	0.2255
GOJQAX	0.1064	0.1765	WAVNAI	0.0551	0.0889
GOZBEC	0.0660	0.1429	WEHTAE	0.0648	0.2119
GULFOI	0.1129	0.1787	**XALSOS**	0.0808	0.1125
**HAMYUP**	0.1216	0.1383	**XAWVUM**	0.0710	0.1040
**HELHOV**	0.0690	0.0958	**XECQEB**	0.0540	0.1164
HELMIU	0.0543	0.2287	XEMNUY	0.1021	0.1723
**HETALA11**	0.0600	0.1263	**XERCAY**	0.1141	0.1245
**HUQBAX**	0.0606	0.1112	XONXUT	0.0427	0.0738
**IDAJON**	0.1064	0.2217	XUJTOL	0.0587	0.1160
IKUWER	0.0815	0.1222	**YUCXAV**	0.0512	0.2157
**KIXHAA**	0.0504	0.1331	**ZAMHEA**	0.0585	0.2756
**LANITA**	0.0379	0.0983	ZAZQAS	0.0694	0.1013
LAPTEB10	0.1106	0.1662	ZEHTUB	0.1072	0.1479
**LIWQEN**	0.0502	0.1240	**ZEJFOJ**	0.0564	0.1001
**MENQOL**	0.0297	0.1359	ZEQVUM	0.0997	0.2726
**MILWEJ**	0.0426	0.1003	ZIDSOX	0.1210	0.1691
NASLUO	0.1577	0.1606	ZIQXIG	0.1029	0.2243
NASTOQ	0.0391	0.0803	ZULFOB	0.0432	0.1534
**NEHDAF**	0.0656	0.0698	**ZUWFOM**	0.0585	0.0812

**Table 8:** Unsigned mean errors, UME_(Gd-L)_s and UMEs, for RM1 model for lanthanides, as compared to the respective experimental crystallographic values, obtained from the Cambridge Structural Database, for each of the 57 cerium(III) complexes.

Structure	RM1		Structure	RM1	
	UME_(Ce-L)_s (Å)	UME (Å)		UME_(Ce-L)_s (Å)	UME (Å)
**ABETEI**	0.0570	0.1694	**JOLYAK**	0.0966	0.1260
ABETUY	0.0508	0.1141	KEDCAX	0.0507	0.1249
AFURUO	0.0709	0.1398	KIXXOE	0.0223	0.0632
APSBCE	0.1310	0.2886	**LELBOT**	0.0838	0.1315
BABZIN	0.0659	0.2240	LIFHUD	0.0492	0.1293
CIBSAH	0.0599	0.1059	**LIKFUH**	0.0649	0.3247
**CUMCIW**	0.0825	0.1876	**MIPTAG**	0.0728	0.1203
DESYAC	0.0843	0.1290	NATCIW	0.1034	0.1928
DEWDEP	0.0736	0.1434	NOJTAH	0.0933	0.1923
**EJIPES**	0.0643	0.1081	OXDACE	0.1151	0.2071
**ETOQUZ**	0.0904	0.1280	PEKWEH	0.0347	0.1101
**ETOROU**	0.1048	0.1430	PIDBAF	0.1492	0.1750
**FEPKAN**	0.0640	0.0734	PUTQAW	0.0986	0.2408
FILKEQ	0.0692	0.0982	**QEGVOO**	0.0789	0.1403
**FOTQOV**	0.1048	0.3579	RIWRAR	0.0281	0.3782
FUHFEZ	0.0494	0.0950	SASCEV	0.1177	0.1556
GACJIE	0.0718	0.1022	**TIJCIY**	0.0779	0.1040
**GAPFIM**	0.1105	0.2786	UKAPEB	0.0460	0.1627
GEGZEX	0.1674	0.1814	ULUQUN	0.0489	0.1076
GETLOG	0.0535	0.0793	VAKJAS	0.0363	0.0972
GINNUM	0.0786	0.1197	VAPCAQ	0.0409	0.1133
HIDLUB	0.1094	0.1252	WAVTAQ	0.1294	0.1695
HIXWEQ	0.0772	0.1228	**WOPHAL**	0.0510	0.1331
HUMDOI	0.0890	0.2701	XASROZ	0.1168	0.1635
HURRAN	0.1177	0.2186	**XEXCUY**	0.1015	0.5062
INDCEP	0.0996	0.1544	XOLMAM	0.0464	0.2421
**JAPPUL**	0.0932	0.2866	XONYAA	0.0601	0.0871
**JEXXOZ**	0.0762	0.2089	ZUNMAW	0.0441	0.0663
JOCCUA	0.0937	0.1357			

**Table 9:** Unsigned mean errors, UME_(Tb-L)_s and UMEs, for RM1 model for lanthanides, as compared to the respective experimental crystallographic values, obtained from the Cambridge Structural Database, [46–48] for each of the 65 praseodymium(III) complexes.

Structure	Method RM1		Structure	Method RM1	
	UME_(Pr-L)_s (Å)	UME (Å)		UME_(Pr-L)_s (Å)	UME (Å)
ACURLB	0.0503	0.1516	KOBRUO	0.0587	0.1110
BABZOT	0.0460	0.1984	LEJSOI	0.0379	0.1816
BAFYOX	0.1280	0.3584	LIYFIJ	0.0583	0.1196
BIFYUK	0.0454	0.2495	**MIPTEK**	0.0966	0.0773
**BUVWIY01**	0.0562	0.1794	**MOGFUJ**	0.0400	0.2012
CAZGUF	0.0290	0.1338	NEPVAF	0.0481	0.1168
**CESROI**	0.0458	0.1909	NEPVUZ	0.0438	0.1765
**CUMCOC**	0.0722	0.1834	NPYPRP10	0.0658	0.1038
DEWDIT	0.0348	0.1324	**PEHHIP**	0.0649	0.1191
DIYMUT	0.0320	0.1600	PEHXIJ	0.0467	0.3069
DORDIX	0.0594	0.2030	PELGOC	0.0459	0.1514
DUCHAK	0.0707	0.1391	POGWIR	0.1623	0.2897
ECABAL	0.0661	0.0767	POPJAF	0.0536	0.2040
EFUJEU	0.0268	0.0683	**PUQNOF**	0.1467	0.1709
EJINUG	0.0330	0.0860	QIMRIN	0.1260	0.2671
**EWIROR**	0.1045	0.3750	QOBBIS	0.0675	0.1509
FAGYIW	0.0857	0.3122	QOVXII	0.0516	0.1505
**FATWOM**	0.0783	0.2212	QOZVEG	0.0591	0.1323
**FEDYAO**	0.0764	0.0899	RASROS	0.0498	0.2181
GIWWEO	0.0764	0.1152	RUGQUF	0.0724	0.3452
GUMXIW	0.1048	0.1463	SERWOB01	0.0383	0.1068
HEDBOH	0.0778	0.2625	VELRUZ	0.0787	0.1039
HEDKAC	0.0648	0.1268	VOXJIB	0.0878	0.1415
HERVUV	0.0810	0.1132	**WUWXAN**	0.0707	0.2963
HODDOT	0.0601	0.2396	**WUWXER**	0.0677	0.1117
HODFEL	0.0763	0.2376	**XASRUF**	0.0540	0.0987
JALMEP	0.0982	0.1785	XAVWUM	0.0664	0.3295
**JALMUF**	0.1256	0.3801	XOKYIF	0.0587	0.1340
**JERWOS**	0.0433	0.2205	XULNUO	0.0731	0.3470
JEXXUF	0.1778	0.2405	YOTYUB	0.0979	0.2103
JUSBII	0.0399	0.1257	ZAXSEW	0.0617	0.2579
**KAHGEF**	0.0766	0.1355	ZULRED	0.0815	0.0805
KAWBIT	0.0170	0.0636			

## Case Study

The new RM1 model was applied to predict the structure of tetramer of praseodymium, [Pr4Cl10(OH)2(thiazole)8(H2O)2][20]. The RM1 structure was calculated using MOPAC 2009 software and keywords used were the following: RM1 (the Hamiltonian used), PRECISE, GNORM = 0.25, SCFCRT = 1.D-10 (in order to increase the SCF convergence criterion) and XYZ (the geometry optimizations were performed in cartesian coordinates).

**Fig 11:**
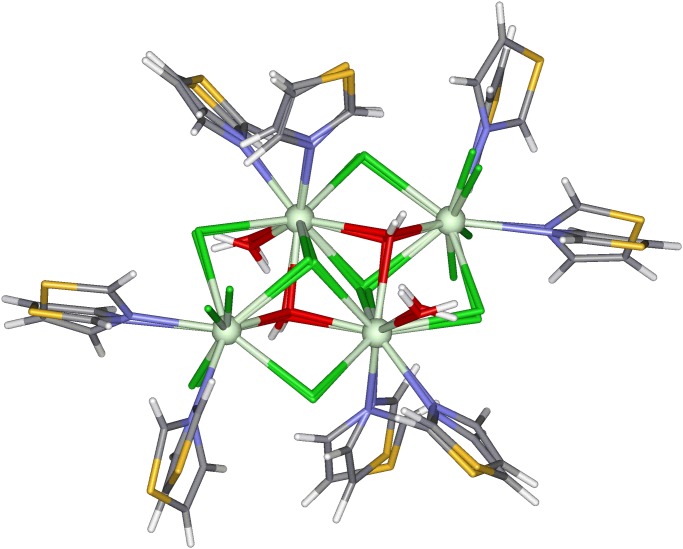
Root mean square deviation fit and alignment of crystallographic and RM1 fully optimized structures of the tetramer of praseodymium, [Pr_4_Cl_10_(OH)_2_(thiazole)_8_(H_2_O)_2_][20].

Fig 11 shows the overlapping of the RM1 and crystallographic structures. The good match observed visually can be confirmed by the low value obtained for the root mean square deviation (RMSD) of 0.034Å, obtained via a RMSD fit and alignment.

A detailed analysis reveals that for the RM1 structure, the average bond length between the Pr3+ ions is 4.54Å whereas the average obtained from crystallographic structure is 4.58Å. The UME considering all Pr3+—L distances (where L = Pr3+, O, N and Cl) is 0.12Å. It is important to highlight that the CPU time for the full geometry optimization using the RM1 model was very fast, less than 3 minutes using a laptop core i7 with 8GB of RAM memory.

## Conclusion

The overall advantage of the RM1 model for the lanthanides presented in this article is that it can perform a full geometry optimization on a complex such as the tetramer of praseodymium, [Pr_4_Cl_10_(OH)_2_(thiazole)_8_(H_2_O)_2_], with relative ease; something that would be exceedingly difficult for an ab initio type calculation. The same can be said of calculations on the three-dimensional 5-aminoisophtalate Pr(III) polymeric complex, which presents good gas storage capabilities [21]. Even if ab initio calculations would be later needed for specific properties that could not be obtained at useful accuracy levels by any other means, they could be carried out on RM1 optimized geometries—something that could save an enormous amount of computing time and resources.

In conclusion, the previous sparkle models seem to be very good models provided the complex has only nitrogen or oxygen directly coordinated to the lanthanide ion. However, if the complex of interest has other types of atoms directly coordinated to the lanthanide ion, then the RM1 model for the lanthanides, introduced in this article, must be the method of choice.

## Supporting Information

S1 Supporting Information Instructions on how to run the RM1 model for the lanthanides in MOPAC2012, together with sample calculations on complexes of each of the parameterized lanthanide trications: La(III), Ce(III), and Pr(III). (DOCX)

## References

[1] Yagyu T, Mizuno Y, Jitsukawa K. Transesterification catalyzed by lanthanum complex grafted upon hydrotalcite as a macroligand. Inorg Chim Acta. 2014; 412: 114–120.

[2] Kobayashi S, Kanai S. Superoxide Scavenging Effects of Some Novel Bis-Ligands and Their Solvated Metal Complexes Prepared by the Reaction of Ligands with Aluminum, Copper and Lanthanum Ions. Molecules. 2013; 18: 6128–6141. 10.3390/molecules18066128 23702919 PMC6270574

[3] Li XY, Shen QC, Zhang GB, Zhang DH, Zheng AQ, Guan F, et al Layered zinc and lanthanum hydroxide nitrates hosting chiral sulphonato-(salen)manganese(III) complex catalyzed asymmetric epoxidation reactions. Catal Commun. 2013; 41: 126–131.

[4] Huang WL, Diaconescu PL. P-4 Activation by Lanthanum and Lutetium Naphthalene Complexes Supported by a Ferrocene Diamide Ligand. Eur J Inorg Chem. 2013; 2013: 4090–4096.

[5] Rastogi RB, Maurya JL, Jaiswal V, Tiwary D. Studies on Lanthanum Complexes of 1-Aryl-2,5-Dithiohydrazodicarbonamides in Paraffin Oil as Extreme Pressure Lubrication Additives. J Tribol-T Asme. 2013; 135.

[6] Jimenez-Reyes M, Solache-Rios M. The influence of pH on the stability constants of lanthanum and europium complexes with humic acids. J Radioanal Nucl Ch. 2012; 293: 273–278.

[7] Akbar R, Baral M, Kanungo BK. Experimental and theoretical approach of photophysical properties of lanthanum(III) and erbium(III) complexes of tris(methoxymethyl)-5-oxine podant. Spectrochim Acta A. 2014; 129: 365–376. 10.1016/j.saa.2014.03.045 24747862

[8] Sharif S, Khan IU, Sahin O, Ahmad S, Buyukgungor O, Ali S. Synthesis and Crystal Structures of a Lanthanum(III) 1D Polymer and a Mixed-Ligand Cerium(III) Binuclear Complex Derived from Pyridine-2,6-dicaboxylic Acid. J Inorg Organomet P. 2012; 22: 1165–1173.

[9] Wang M, Yu L, Li FZ, Xie JQ. The interactions between an aza-cyclic cerium (III) complex and pUC19 DNA. Chinese J Catal. 2014; 35: 524–531.

[10] Huang C, Cai SL, Zou LK, Feng JS, Xie JQ, Xie B. Kinetic Study of the Hydrolysis of BNPP by the Cerium(III) Complex. J Disper Sci Technol. 2012; 33: 1292–1296.

[11] Park J, Kim D. Effect of cerium/18-crown-6-ether coordination complex OH center dot quencher on the properties of sulfonated poly(ether ether ketone) fuel cell electrolyte membranes. J Membrane Sci. 2014; 469: 238–244.

[12] Alghool S, Abd El-Halim HF, Abd El-Sadek MS, Yahia IS, Wahab LA. Synthesis, thermal characterization, and antimicrobial activity of lanthanum, cerium, and thorium complexes of amino acid Schiff base ligand. J Therm Anal Calorim. 2013; 112: 671–681.

[13] Zahir MH. Synthesis and Characterization of Trivalent Cerium Complexes of p-tert-Butylcalix[4,6,8]Arenes: Effect of Organic Solvents. J Chem-Ny. 2013.

[14] Pawlicki G, Lis S. Luminescence study of praseodymium complexes with selected phosphonate ligands. Opt Mater. 2011; 33: 1544–1547.

[15] Hong ZR, Liang CJ, Li RG, Zang FX, Fan D, Li WL, et al Infrared and visible emission from organic electroluminescent devices based on praseodymium complex. Appl Phys Lett. 2001; 79: 1942–1944.

[16] Khorasani-Motlagh M, Noroozifar M, Mirkazehi-Rigi S. Fluorescence and DNA-binding properties of neodymium(III) and praseodymium(III) complexes containing 1,10-phenanthroline. Spectrochim Acta A. 2011; 79: 978–984. 10.1016/j.saa.2011.04.009 21669548

[17] Li CH, Song XZ, Jiang JH, Gu HW, Tao LM, Yang P, et al Synthesis, crystal structure and thermodynamic properties of a new praseodymium Schiff-base complex. Thermochim Acta. 2014; 581: 118–122.

[18] Zhao J, Tang WW, Liang JJ, Zhang Y, Jia DX. Neutral Praseodymium(III) Complexes with Tetraselenidoantimonate Ligand: Coligand Influence of Mixed Polyamines on the Coordination Mode of [SbSe4](3-) Anion. J Chem Crystallogr. 2012; 42: 187–191.

[19] Pevec A, Mrak M, Demsar A, Petricek S, Roesky HW. Coordination number 12 in praseodymium and 11 in neodymium complexes with organofluorotitanate ligands. Polyhedron. 2003; 22: 575–579.

[20] Dannenbauer N, Muller-Buschbaum K. An Oxygen/Chlorine Double-Capped Praseodymium Tetramer Cluster Complex with Thiazole. Z Anorg Allg Chem. 2013; 639: 2737–2740.

[21] Qiu YC, Deng H, Yang SH, Mou JX, Daiguebonne C, Kerbellec N, et al Syntheses, Crystal Structures, and Gas Storage Studies in New Three-Dimensional 5-Aminoisophthalate Praseodymium Polymeric Complexes. Inorg Chem. 2009; 48: 3976–3981. 10.1021/ic8020518 19351164

[22] de Andrade AVM, da Costa NB, Simas AM, de Sa GF. Sparkle Model for the Quantum-Chemical Am1 Calculation of Europium Complexes. Chem Phys Lett. 1994; 227: 349–353.

[23] de Andrade AVM, da Costa NB, Simas AM, de Sa GF. Sparkle Model for the Quantum-Chemical Am1 Calculation of Europium Complexes of Coordination-Number-9. J Alloy Compd. 1995; 225: 55–59.

[24] Dewar MJS, Zoebisch EG, Healy EF, Stewart JJP. The Development and Use of Quantum-Mechanical Molecular-models. 76. AM1—A New General-Purpose Quantum-Mechanical Molecular-Model. J Am Chem Soc. 1985; 107: 3902–3909.

[25] Ridley JE, Zerner MC. Triplet-States Via Intermediate Neglect of Differential Overlap—Benzene, Pyridine and Diazines. Theor Chim Acta. 1976; 42: 223–236.

[26] de Andrade AVM, Longo RL, Simas AM, de Sa GF. Theoretical model for the prediction of electronic spectra of lanthanide complexes. J Chem Soc Faraday T. 1996; 92: 1835–1839.

[27] da Costa NB, Freire RO, dos Santos MAC, Mesquita ME. Sparkle model and intensity parameters of the Eu(3-amino-2-carboxypyridine-N-oxide)(3)3H2O complex. J Mol Struc-Theochem. 2001; 545: 131–135.

[28] de Mesquita ME, Junior SA, Oliveira FC, Freire RO, Junior NBC, de Sa GF. Synthesis, spectroscopic studies and structure prediction of the new Tb(3-NH2PIC)(3)center dot 3H(2)O complex. Inorg Chem Commun. 2002; 5: 292–295.

[29] Faustino WM, Rocha GB, Silva FRGE, Malta OL, de Sa GF, Simas AM. Design of ligands to obtain lanthanide ion complexes displaying high quantum efficiencies of luminescence using the sparkle model. J Mol Struc-Theochem. 2000; 527: 245–251.

[30] Freire RO, Silva FRGE, Rodrigues MO, de Mesquita ME, Junior NBD. Design of europium(III) complexes with high quantum yield. J Mol Model. 2005; 12: 16–23. 16044288 10.1007/s00894-005-0280-7

[31] dos Santos ER, dos Santos MAC, Freire RO, Junior SA, Barreto LS, de Mesquita ME. On the use of theoretical tools in the study of photophysical properties of the new Eu(fod)(3) complex with diphenbipy. Chem Phys Lett. 2006; 418: 337–341.

[32] dos Santos ER, Freire RO, da Costa NB, Paz FAA, de Simone CA, Junior SA, et al Theoretical and Experimental Spectroscopic Approach of Fluorinated Ln(3+)-beta-Diketonate Complexes. J Phys Chem A. 2010; 114: 7928–7936. 10.1021/jp104038r 20617802

[33] Freire RO, Rocha GB, Albuquerque RQ, Simas AM. Efficacy of the semiempirical sparkle model as compared to ECP ab-initio calculations for the prediction of ligand field parameters of europium(III) complexes. J Lumin. 2005; 111: 81–87.

[34] Rocha GB, Freire RO, da Costa NB, de Sá GF, Simas AM. Sparkle Model for AM1 Calculation of Lanthanide Complexes: Improved Parameters for Europium. 2004; 43: 2346–2354. 10.1021/ic034882p 15046511

[35] Freire RO, Rocha GB, Simas AM. Sparkle model for the calculation of lanthanide complexes: AM1 parameters for Eu(III), Gd(III), and Tb(III). Inorg Chem. 2005; 44: 3299–3310. 15847440 10.1021/ic048530+

[36] da Costa NB, Freire RO, Simas AM, Rocha GB. Structure modeling of trivalent lanthanum and lutetium complexes: Sparkle/PM3. J Phys Chem A. 2007; 111: 5015–5018. 17506532 10.1021/jp0672104

[37] Simas AM, Freire RO, Rocha GB. Cerium (III) complexes modeling with Sparkle/PM3. Lect Notes Comput Sc. 2007; 4488: 312–318.

[38] Freire RO, Rocha GB, Simas AM. Sparkle/PM3 parameters for praseodymium(III) and ytterbium(III). Chem Phys Lett. 2007; 441: 354–357.

[39] Freire RO, Simas AM. Sparkle/PM6 Parameters for all Lanthanide Trications from La(III) to Lu(III). J Chem Theory Comput. 2010; 6: 2019–2023. 26615930 10.1021/ct100192c

[40] Dutra JDL, Filho MAM, Rocha GB, Freire RO, Simas AM, Stewart JJP. Sparkle/PM7 Lanthanide Parameters for the Modeling of Complexes and Materials. 2013; 9: 3333–3341. 10.1021/ct301012h PMC3806451 24163641

[41] Filho MAM, Dutra JDL, Rocha GB, Freire RO, Simas AM. Sparkle/RM1 parameters for the semiempirical quantum chemical calculation of lanthanide complexes. 2013; 3: 16747–16755.

[42] Filho MA, Dutra JDL, Cavalcanti HL, Rocha GB, Simas AM, Freire RO. RM1 Model for the Prediction of Geometries of Complexes of the Trications of Eu, Gd, and Tb. 2014; 10: 3031–3037. 10.1021/ct400909w 26588274

[43] Filho MAM, Dutra JDL, Rocha GB, Simas AM, Freire RO. Semiempirical Quantum Chemistry Model for the Lanthanides: RM1 (Recife Model 1) Parameters for Dysprosium, Holmium and Erbium. 2014; 9: e86376. 10.1371/journal.pone.0086376 PMC3908927 24497945

[44] Rocha GB, Freire RO, Simas AM, Stewart JJP. RM1: A reparameterization of AM1 for H, C, N, O, P, S, F, Cl, Br, and I. J Comput Chem. 2006; 27: 1101–1111. 16691568 10.1002/jcc.20425

[45] Freire RO, Rocha GB, Simas AM. Modeling rare earth complexes: Sparkle/PM3 parameters for thulium(III). Chem Phys Lett. 2006; 425: 138–141.

[46] Allen FH. The Cambridge Structural Database: a quarter of a million crystal structures and rising. Acta Crystallogr B. 2002; 58: 380–388. 12037359 10.1107/s0108768102003890

[47] Allen FH, Motherwell WDS. Applications of the Cambridge Structural Database in organic chemistry and crystal chemistry. Acta Crystallogr B. 2002; 58: 407–422. 12037362 10.1107/s0108768102004895

[48] Bruno IJ, Cole JC, Edgington PR, Kessler M, Macrae CF, McCabe P, et al New software for searching the Cambridge Structural Database and visualizing crystal structures. Acta Crystallogr B. 2002; 58: 389–397. 12037360 10.1107/s0108768102003324

[49] Freire RO, da Costa NB, Rocha GB, Simas AM. Sparkle/AM1 structure modeling of lanthanum (III) and lutetium (III) complexes. J Phys Chem A. 2006; 110: 5897–5900. 16640387 10.1021/jp057286k

[50] Kaufman L, Rousseeuw PJ (2009) Finding groups in data: an introduction to cluster analysis: John Wiley & Sons.

