# Supplementary material for: RM1 Semiempirical Quantum Chemistry: Parameters for Trivalent Lanthanum, Cerium and Praseodymium
Source: PLoS One. 2015 Jul 1;10(7):e0124372. doi: 10.1371/journal.pone.0124372 (PMC4489505; doi:10.1371/journal.pone.0124372)
Supplement: S1 Supporting Information — (DOCX) [file pone.0124372.s001.docx]

RM1 Semiempirical Quantum Chemistry: Parameters for Trivalent Lanthanum, Cerium and Praseodymium

Supporting Information

José Diogo L. Dutra^1,3^, Manoel A. M. Filho^1^, Gerd B. Rocha^2^,

Alfredo M. Simas^3^_,_ and Ricardo O. Freire^1*^

^1^Pople Computational Chemistry Laboratory, Departamento de Química, Universidade Federal de Sergipe, 49.100-000 – São Cristóvão, SE, Brazil.

^2^Departamento de Química, CCEN, Universidade Federal da Paraíba, 58.059-970 – João Pessoa, PB, Brazil.

^3^Departamento de Química Fundamental, Universidade Federal de Pernambuco, 50.740-540, Recife, PE, Brazil.

^*^Corresponding author. e-mail: [rfreire@ufs.br](mailto:rfreire@ufs.br)

Contents

| 1. [How to run lanthanide complexes RM1 calculations with MOPAC2012.](#link13) ………………. | 2 |
| --- | --- |
| 1. [MOPAC2012 Input (.mop) and output (.arc) files.](#link3) ……………………………………………….…….. | 4 |
| 1. [Graphical User Interfaces for MOPAC2012](#link4) ………………………………………………………………. | 4 |
| 1. [Sample Input and Output Files](#link6)……………………………………………………………………..…………… | 5 |

1. **How to run lanthanide complexes RM1 calculations with MOPAC2012**

([back to contents](#Contents))

MOPAC2012 is the new software released by Prof. James J. P. Stewart from *Stewart Computational Chemistry* of Colorado Springs, CO, and represents the most recent version of the MOPAC series of molecular modeling softwares, which started in 1981.

MOPAC2012 has Sparkle/AM1, Sparkle/PM3, Sparkle/PM6, Sparkle/PM7, and Sparkle/RM1 fully implemented. Instructions on how to use the Sparkle Model in MOPAC2012, and on how to visualize the complexes with graphical user interfaces, can be found at <http://www.sparkle.pro.br>.

A MOPAC2012 executable can be obtained from <http://openmopac.net> and is presently free for academics.

In order to be acquainted with the software, users are encouraged to read the MOPAC2012 manual at <http://openmopac.net/manual/>.

As the MOPAC2012 manual says:

*MOPAC is written with the non-theoretician in mind.*

*While MOPAC calls upon many concepts in quantum theory and thermodynamics and uses some fairly advanced mathematics, the users need not be familiar with these specialized topics.*

At present, the most recent version of MOPAC2012 is 12.236W.

To run a RM1 model for lanthanides calculation in MOPAC 2012, proceed as follows:

1. Create a data-file with extension .mop which describes a molecular system and specifies the type of calculation that is to be carried out.
   1. Use only the keyword [RM1](http://openmopac.net/manual/rm1_key.html). Do not forget to set the charge n of the complex with keyword [CHARGE=n](http://openmopac.net/manual/charge.html)

To run a Sparkle/RM1 calculation in MOPAC 2012, proceed as follows:

- 1. Use the lanthanide as you would use any atom in MOPAC.
  2. Do not forget to set the charge n of the complex with keyword [CHARGE=n](http://openmopac.net/manual/charge.html)
  3. For a Sparkle/RM1 calculation, use the keywords [RM1](http://openmopac.net/manual/am1.html) [SPARKLE](http://openmopac.net/manual/sparkle.html) in the keyword line.

1. Command MOPAC to run the calculation using that data-file.
2. Get the desired output on the system from the output files created by MOPAC.
3. **MOPAC2012 Input (.mop) and output (.arc) files** ([back to contents](#Contents))

Sample input and output files for all Sparkle Models can be found in <http://www.sparkle.pro.br>.

As examples, we are providing in the appendix of this supplementary material the content of a MOPAC2012 input and the corresponding RM1 output file for one complex for each lanthanide ion.

In order to reproduce the calculation, please [request a password and download](http://openmopac.net/download-c.html) MOPAC2012.exe from <http://openmopac.net>, which is presently free for academics. Then, copy the contents of one of the sample inputs to a text file, name it something like sample.mop, and simply open it with MOPAC2012.

Warning: MOPAC2012 output files with extension .arc may be confused with some types of compressed files in some Windows systems. Be sure to open them with notepad, or a similar text editor.

*At present, this supplementary material shows output files from a version of MOPAC2009 we used to implement the RM1 model for lanthanides. As soon as the paper is accepted, this model will be implemented in MOPAC2012 and this supplementary information will be updated accordingly.*

1. **Graphical User Interfaces for MOPAC2012** ([back to contents](#Contents))

A large number of graphical user interfaces, GUIs, that can be used with MOPAC2012, both commercial and free, can be found [here](http://openmopac.net/resellers.html).

Warning: the bond connection algorithm of some of the Graphical User Interfaces may not work efficiently with some high coordination number lanthanide complexes. Some coordinating bonds may not appear, while sometimes some other spurious bond connections may also appear. However, the positions of the atoms are always correct.

1. **Sample Input and Output Files** ([back to contents](#Contents))


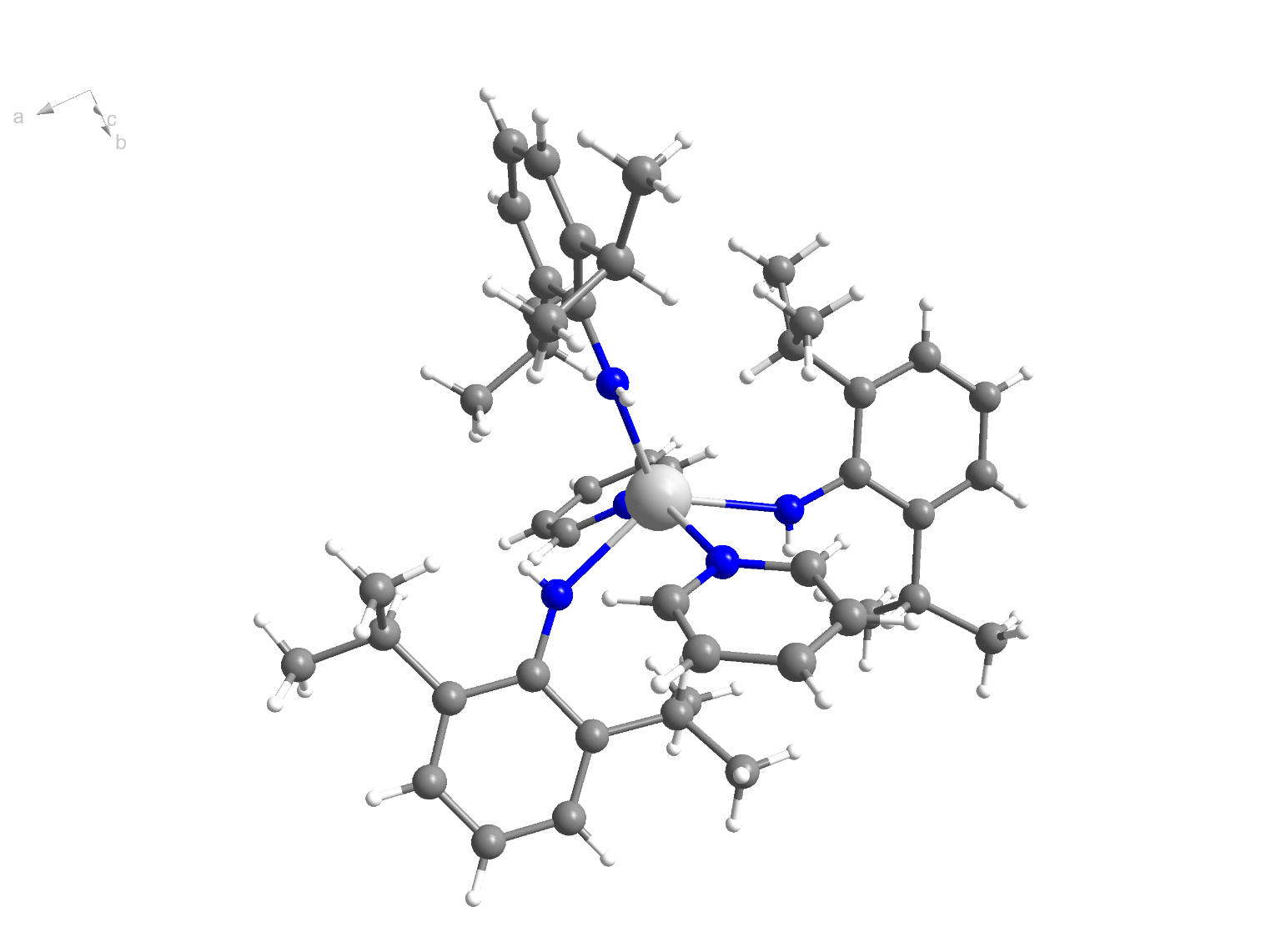


S1_Figura: Crystallographic structure of the Lanthanum complex (EZIPUY)

---------------------------------------- Begin of file **EZIPUY.mop**-----------------------------------

RM1 EXTERNAL=spk.inp PRECISE NOINTER XYZ T=10D NOLOG GEO-OK +

BFGS GNORM=0.25 SCFCRT=1.D-10 CYCLES=5000 CHARGE=0.0

Coordination Number: 5, Compound Type: 1

La 0.0000 1 0.0000 1 0.0000 1

N -0.0765 1 -2.3622 1 0.1536 1

N 2.2291 1 0.5404 1 0.6151 1

N -1.9810 1 1.3560 1 -0.0853 1

N -0.4428 1 0.0236 1 2.7012 1

N 0.5202 1 1.2002 1 -2.3778 1

C -0.1173 1 -3.5827 1 -0.5300 1

C 0.2070 1 -3.6124 1 -1.9107 1

C 0.6883 1 -2.3353 1 -2.5748 1

H 0.1484 1 -1.6033 1 -2.2077 1

C 0.5250 1 -2.2886 1 -4.0952 1

H 0.8585 1 -1.4518 1 -4.4272 1

H -0.4040 1 -2.3749 1 -4.3191 1

H 1.0193 1 -3.0107 1 -4.4920 1

C 2.1429 1 -2.0606 1 -2.1743 1

H 2.4466 1 -1.2564 1 -2.6025 1

H 2.6950 1 -2.7969 1 -2.4493 1

H 2.1992 1 -1.9572 1 -1.2217 1

C 0.1552 1 -4.8089 1 -2.6025 1

H 0.3575 1 -4.8159 1 -3.5099 1

C -0.1900 1 -5.9883 1 -1.9818 1

H -0.2066 1 -6.7840 1 -2.4611 1

C -0.5088 1 -5.9742 1 -0.6462 1

H -0.7469 1 -6.7699 1 -0.2298 1

C -0.8189 1 -4.8428 1 1.5978 1

H -1.3531 1 -4.0443 1 1.7971 1

C -1.6266 1 -6.0435 1 2.0328 1

H -2.4359 1 -6.0888 1 1.5182 1

H -1.8450 1 -5.9614 1 2.9638 1

H -1.1136 1 -6.8421 1 1.8934 1

C 0.4675 1 -4.7508 1 2.4315 1

H 0.9652 1 -3.9735 1 2.1684 1

H 0.9990 1 -5.5366 1 2.2862 1

H 0.2412 1 -4.6857 1 3.3625 1

C -0.4853 1 -4.8060 1 0.0958 1

C 3.1386 1 1.3517 1 1.2927 1

C 6.5679 1 -0.2681 1 0.5224 1

H 6.8115 1 0.5135 1 0.0216 1

H 6.8131 1 -1.0539 1 0.0294 1

H 7.0252 1 -0.2638 1 1.3670 1

C 4.5236 1 1.0034 1 1.4112 1

C 5.0493 1 -0.2681 1 0.7642 1

H 4.6136 1 -0.3530 1 -0.1100 1

C 4.6564 1 -1.4857 1 1.5850 1

H 4.9889 1 -2.2787 1 1.1608 1

H 3.7004 1 -1.5339 1 1.6479 1

H 5.0321 1 -1.4121 1 2.4649 1

C 5.3613 1 1.8558 1 2.1238 1

H 6.2647 1 1.6462 1 2.1860 1

C 4.8996 1 2.9970 1 2.7395 1

H 5.4797 1 3.5308 1 3.2309 1

C 3.5740 1 3.3382 1 2.6207 1

H 3.2681 1 4.1170 1 3.0267 1

C 1.2472 1 2.9772 1 1.7386 1

H 0.7188 1 2.1574 1 1.6439 1

C 2.6748 1 2.5453 1 1.9059 1

C 1.0529 1 3.7715 1 0.4360 1

H 0.1297 1 4.0235 1 0.3515 1

H 1.3059 1 3.2264 1 -0.3123 1

H 1.5998 1 4.5601 1 0.4576 1

C 0.6538 1 3.7375 1 2.9304 1

H -0.2615 1 3.9626 1 2.7438 1

H 1.1568 1 4.5403 1 3.0797 1

H 0.6923 1 3.1839 1 3.7141 1

C -3.3332 1 1.4409 1 -0.3948 1

C -3.9923 1 0.3167 1 -0.9399 1

C -3.2387 1 -0.9888 1 -1.1235 1

H -2.2892 1 -0.7636 1 -1.2060 1

C -3.3838 1 -1.8680 1 0.1158 1

H -2.9044 1 -2.6893 1 -0.0157 1

H -4.3124 1 -2.0606 1 0.2651 1

H -3.0267 1 -1.4079 1 0.8799 1

C -3.6229 1 -1.7888 1 -2.3766 1

H -3.0953 1 -2.5902 1 -2.4179 1

H -3.4572 1 -1.2564 1 -3.1583 1

H -4.5532 1 -2.0195 1 -2.3373 1

C -5.3477 1 0.3818 1 -1.2494 1

H -5.7629 1 -0.3658 1 -1.6145 1

C -6.0945 1 1.5230 1 -1.0312 1

H -6.9987 1 1.5457 1 -1.2414 1

C -5.4649 1 2.6402 1 -0.4924 1

H -5.9656 1 3.4062 1 -0.3280 1

C -4.3596 1 4.8915 1 1.0193 1

H -3.8605 1 5.6546 1 1.3159 1

H -4.7671 1 4.4624 1 1.7736 1

H -5.0417 1 5.1746 1 0.4046 1

C -4.1065 1 2.6430 1 -0.1904 1

C -2.6988 1 4.6111 1 -0.8407 1

H -2.2636 1 5.4040 1 -0.5166 1

H -3.3344 1 4.8518 1 -1.5183 1

H -2.0427 1 4.0179 1 -1.2139 1

C -3.4236 1 3.9117 1 0.3201 1

H -2.7439 1 3.6384 1 0.9722 1

C -1.5989 1 0.4583 1 3.2290 1

H -2.3161 1 0.6353 1 2.6653 1

C -1.7518 1 0.6480 1 4.5940 1

H -2.5609 1 0.9609 1 4.9299 1

C -0.7228 1 0.3804 1 5.4425 1

H -0.8155 1 0.5022 1 6.3598 1

C 0.4631 1 -0.0783 1 4.9044 1

H 1.1875 1 -0.2752 1 5.4543 1

C 0.5585 1 -0.2369 1 3.5511 1

H 1.3643 1 -0.5428 1 3.1995 1

C -0.4453 1 1.3999 1 -3.2762 1

H -1.3273 1 1.2738 1 -3.0091 1

C -0.2042 1 1.7836 1 -4.5784 1

H -0.9070 1 1.9195 1 -5.1735 1

C 1.1020 1 1.9606 1 -4.9752 1

H 1.2932 1 2.1942 1 -5.8551 1

C 2.1087 1 1.7921 1 -4.0834 1

H 2.9947 1 1.9280 1 -4.3329 1

C 1.7851 1 1.4112 1 -2.7871 1

H 2.4735 1 1.2965 1 -2.1724 1

H -0.1755 1 -2.3778 1 0.9133 1

H 2.5139 1 0.0293 1 0.2455 1

H -1.7217 1 2.0115 1 0.0687 1

0---------------------------------------- End of file **EZIPUY.mop**-----------------------------------

---------------------------------------- Begin of file **EZIPUY.arc**-----------------------------------

SUMMARY OF RM1 CALCULATION, Site No: 999

MOPAC2009 (Version: 11.03W )

Wed Jun 25 19:25:32 2014

Empirical Formula: C46 H64 N5 La = 116 atoms

RM1 EXTERNAL=spk.inp PRECISE NOINTER XYZ T=10D NOLOG GEO-OK +

BFGS GNORM=0.25 SCFCRT=1.D-10 CYCLES=5000 CHARGE=0.0

Coordination Number: 5, Compound Type: 1

PETERS TEST WAS SATISFIED IN BFGS OPTIMIZATION

SCF FIELD WAS ACHIEVED

HEAT OF FORMATION = -381.81625 KCAL/MOL = -1597.51918 KJ/MOL

TOTAL ENERGY = -7776.03408 EV

ELECTRONIC ENERGY = -105147.98769 EV

CORE-CORE REPULSION = 97371.95361 EV

GRADIENT NORM = 0.22428

DIPOLE = 0.61596 DEBYE POINT GROUP: C1

NO. OF FILLED LEVELS = 138

IONIZATION POTENTIAL = 7.486216 EV

HOMO LUMO ENERGIES (EV) = -7.486 -1.108

MOLECULAR WEIGHT = 825.951

COSMO AREA = 656.63 SQUARE ANGSTROMS

COSMO VOLUME = 1025.77 CUBIC ANGSTROMS

MOLECULAR DIMENSIONS (Angstroms)

Atom Atom Distance

H 30 H 51 13.87686

H 109 H 99 13.18687

H 80 H 39 12.94593

SCF CALCULATIONS = 961

COMPUTATION TIME = 1 HOURS 11 MINUTES AND 13.741 SECONDS

FINAL GEOMETRY OBTAINED CHARGE

RM1 EXTERNAL=spk.inp PRECISE NOINTER XYZ T=10D NOLOG GEO-OK +

BFGS GNORM=0.25 SCFCRT=1.D-10 CYCLES=5000 CHARGE=0.0

Coordination Number: 5, Compound Type: 1

La 0.03703952 +1 -0.07756283 +1 0.09524343 +1 -0.0895

N -0.14101591 +1 -2.41436487 +1 -0.20340256 +1 -0.4108

N 2.23928517 +1 0.60747953 +1 0.63262115 +1 -0.4022

N -1.88827869 +1 1.34102987 +1 0.09296950 +1 -0.4285

N -0.50778706 +1 -0.49340968 +1 2.74016162 +1 -0.1978

N 0.29054126 +1 0.64585597 +1 -2.52761635 +1 -0.1932

C 0.35793820 +1 -3.69747380 +1 -0.52460800 +1 0.1427

C 1.51552840 +1 -3.77923082 +1 -1.32947302 +1 -0.1163

C 2.22809408 +1 -2.56130938 +1 -1.84878804 +1 0.0369

H 1.79141479 +1 -1.59410252 +1 -1.42471003 +1 0.0857

C 2.09597331 +1 -2.50902484 +1 -3.37200310 +1 -0.1824

H 2.62357943 +1 -1.64268562 +1 -3.79717614 +1 0.0531

H 1.04484000 +1 -2.43240867 +1 -3.68671630 +1 0.0597

H 2.50733693 +1 -3.39708423 +1 -3.87215397 +1 0.0640

C 3.70618462 +1 -2.61754094 +1 -1.45671515 +1 -0.1936

H 4.20694723 +1 -1.64831299 +1 -1.60168846 +1 0.0588

H 4.28286429 +1 -3.34486934 +1 -2.04596163 +1 0.0636

H 3.83960333 +1 -2.89818632 +1 -0.40196387 +1 0.0664

C 2.02038478 +1 -5.02505236 +1 -1.67971347 +1 -0.0629

H 2.91108278 +1 -5.11127431 +1 -2.31009620 +1 0.1027

C 1.40913072 +1 -6.19138707 +1 -1.24305800 +1 -0.1631

H 1.81858854 +1 -7.16454322 +1 -1.52613758 +1 0.1053

C 0.27768308 +1 -6.11708711 +1 -0.44536757 +1 -0.0546

H -0.18651836 +1 -7.05029179 +1 -0.11114383 +1 0.1022

C -1.47633522 +1 -4.89168822 +1 0.79552570 +1 -0.0007

H -1.80358714 +1 -3.84107577 +1 1.02997837 +1 0.0609

C -2.63349244 +1 -5.58745350 +1 0.08273688 +1 -0.1842

H -2.81776126 +1 -5.15914891 +1 -0.91306040 +1 0.0630

H -3.57216239 +1 -5.49711637 +1 0.64917934 +1 0.0555

H -2.46620460 +1 -6.66322440 +1 -0.06604511 +1 0.0636

C -1.17378662 +1 -5.58123769 +1 2.12353540 +1 -0.1792

H -0.30620236 +1 -5.13217267 +1 2.62753574 +1 0.0621

H -0.95444546 +1 -6.65262404 +1 2.01670458 +1 0.0625

H -2.02221022 +1 -5.50899519 +1 2.81985308 +1 0.0537

C -0.25603635 +1 -4.88830257 +1 -0.07805712 +1 -0.1462

C 3.21595778 +1 1.55586872 +1 1.01156640 +1 0.1364

C 6.19359272 +1 -0.28828416 +1 0.18346371 +1 -0.1825

H 5.89207284 +1 0.12893023 +1 -0.78806816 +1 0.0626

H 6.49083538 +1 -1.33240692 +1 0.00527733 +1 0.0573

H 7.10660928 +1 0.24435838 +1 0.48353000 +1 0.0629

C 4.55771235 +1 1.21547357 +1 1.29289194 +1 -0.1449

C 5.07255514 +1 -0.19170389 +1 1.21543567 +1 -0.0031

H 4.25678851 +1 -0.89771278 +1 0.89686349 +1 0.0625

C 5.56530599 +1 -0.65123158 +1 2.58530308 +1 -0.1803

H 5.86253035 +1 -1.71027239 +1 2.57428184 +1 0.0570

H 4.78808502 +1 -0.54485198 +1 3.35537357 +1 0.0597

H 6.43952188 +1 -0.08971401 +1 2.94282714 +1 0.0631

C 5.45579029 +1 2.20734548 +1 1.66672144 +1 -0.0544

H 6.50038396 +1 1.96187582 +1 1.88333759 +1 0.1032

C 5.05246963 +1 3.52901699 +1 1.78091338 +1 -0.1620

H 5.76778137 +1 4.29790317 +1 2.08393894 +1 0.1061

C 3.73480599 +1 3.87010916 +1 1.51419052 +1 -0.0634

H 3.43071833 +1 4.91533333 +1 1.63195803 +1 0.1042

C 1.39710997 +1 3.33237110 +1 0.86702901 +1 0.0381

H 0.84693958 +1 2.56395447 +1 0.22257915 +1 0.0762

C 2.81654030 +1 2.90757422 +1 1.11702662 +1 -0.1203

C 1.32935129 +1 4.66105277 +1 0.11260763 +1 -0.1931

H 0.30856533 +1 4.88144497 +1 -0.23384154 +1 0.0599

H 1.97666385 +1 4.66133224 +1 -0.77574819 +1 0.0627

H 1.63133955 +1 5.52187514 +1 0.72665027 +1 0.0660

C 0.69868816 +1 3.45904909 +1 2.22268497 +1 -0.1860

H -0.32500970 +1 3.85255400 +1 2.13571066 +1 0.0594

H 1.23605538 +1 4.13948330 +1 2.90002782 +1 0.0676

H 0.62878907 +1 2.48917225 +1 2.74206073 +1 0.0716

C -3.26116727 +1 1.59664800 +1 -0.07677329 +1 0.1602

C -4.09359862 +1 0.54125919 +1 -0.51569767 +1 -0.1385

C -3.54675169 +1 -0.80348842 +1 -0.90191418 +1 0.0395

H -2.40673860 +1 -0.79036468 +1 -0.95912786 +1 0.0804

C -3.97503205 +1 -1.84307757 +1 0.13464029 +1 -0.1894

H -3.70850516 +1 -2.86616768 +1 -0.17188551 +1 0.0608

H -5.06047233 +1 -1.84972443 +1 0.30902442 +1 0.0674

H -3.50143103 +1 -1.66201033 +1 1.11235032 +1 0.0583

C -4.05489249 +1 -1.20754971 +1 -2.28835358 +1 -0.1913

H -3.51648232 +1 -2.08341650 +1 -2.68040658 +1 0.0555

H -3.93073555 +1 -0.39813793 +1 -3.02177027 +1 0.0633

H -5.11965829 +1 -1.47991959 +1 -2.29688994 +1 0.0651

C -5.46626617 +1 0.73376446 +1 -0.59849007 +1 -0.0529

H -6.12714362 +1 -0.08043756 +1 -0.91283766 +1 0.1006

C -6.03596605 +1 1.95767434 +1 -0.28044036 +1 -0.1783

H -7.11932814 +1 2.09024770 +1 -0.33377171 +1 0.1053

C -5.22097909 +1 3.01507387 +1 0.09451167 +1 -0.0444

H -5.68899923 +1 3.97743083 +1 0.32538985 +1 0.1005

C -3.29121617 +1 4.45871346 +1 1.99316508 +1 -0.1810

H -2.68332628 +1 5.32317780 +1 2.29820234 +1 0.0558

H -3.05140992 +1 3.63987116 +1 2.68653562 +1 0.0576

H -4.33937791 +1 4.73113112 +1 2.17987320 +1 0.0636

C -3.84405939 +1 2.86038923 +1 0.17983374 +1 -0.1652

C -3.34662922 +1 5.23492578 +1 -0.39106022 +1 -0.1818

H -2.63530487 +1 6.06331946 +1 -0.25626437 +1 0.0541

H -4.34584240 +1 5.66318861 +1 -0.23107836 +1 0.0616

H -3.29572027 +1 4.93805665 +1 -1.44817513 +1 0.0646

C -3.02996194 +1 4.06758019 +1 0.54160172 +1 0.0016

H -1.93199660 +1 3.85163074 +1 0.42777988 +1 0.0600

C -1.71888722 +1 -0.19693975 +1 3.30274585 +1 0.0627

H -2.48649198 +1 0.23031797 +1 2.62838577 +1 0.1633

C -2.01072437 +1 -0.40750538 +1 4.65244207 +1 -0.1839

H -2.99880142 +1 -0.14867584 +1 5.04334801 +1 0.1359

C -1.03373341 +1 -0.94493486 +1 5.48026974 +1 -0.0065

H -1.23715360 +1 -1.11855898 +1 6.54189644 +1 0.1191

C 0.20616637 +1 -1.25826134 +1 4.93827939 +1 -0.1841

H 0.99954260 +1 -1.68345895 +1 5.55936355 +1 0.1355

C 0.43403722 +1 -1.02298391 +1 3.58059644 +1 0.0764

H 1.41666545 +1 -1.26744855 +1 3.13354720 +1 0.1537

C -0.52062630 +1 0.13132903 +1 -3.50101540 +1 0.0741

H -1.26240703 +1 -0.62631553 +1 -3.18114199 +1 0.1552

C -0.45058501 +1 0.51688102 +1 -4.84230253 +1 -0.1819

H -1.12876567 +1 0.06980630 +1 -5.57460479 +1 0.1354

C 0.48473699 +1 1.46917809 +1 -5.22443797 +1 -0.0076

H 0.55874446 +1 1.78976104 +1 -6.26861221 +1 0.1193

C 1.32550164 +1 2.00820353 +1 -4.25876795 +1 -0.1812

H 2.07560185 +1 2.75895254 +1 -4.52284610 +1 0.1352

C 1.20422382 +1 1.57949463 +1 -2.93545421 +1 0.0632

H 1.86815500 +1 1.99789304 +1 -2.15448739 +1 0.1571

H -1.13897004 +1 -2.49277661 +1 0.12275741 +1 0.1900

H 2.67734306 +1 -0.34293103 +1 0.52512130 +1 0.1899

H -1.38734574 +1 2.22213069 +1 0.35473641 +1 0.1825

---------------------------------------- End of file **EZIPUY.arc**-----------------------------------

**
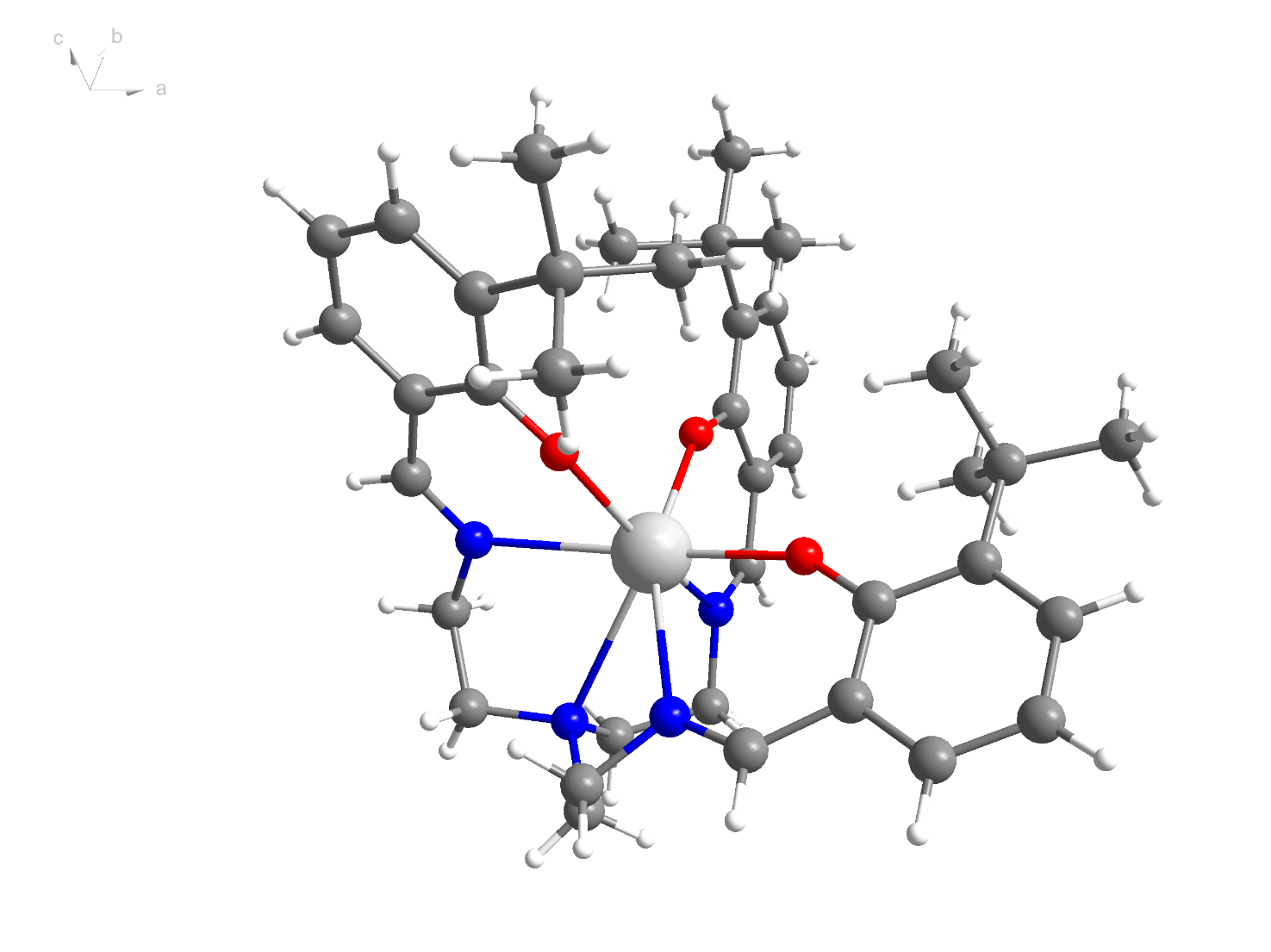
**

S2_Figure: Crystallographic structure of the Cerium complex (GACJIE)

---------------------------------------- Begin of file **GACJIE.mop**-----------------------------------

RM1 EXTERNAL=spk.inp PRECISE NOINTER XYZ BFGS T=10D GNORM=0.25 +

NOLOG GEO-OK SCFCRT=1.D-10 charge=0

NUMERO DE COORDENAÇÃO = 8

Ce 0.000000 1 0.000000 1 0.000000 1

O 2.277100 1 0.000000 1 0.000000 1

O -0.072690 1 2.283443 1 0.000000 1

O -0.445083 1 -0.184346 1 2.228524 1

N -1.466474 1 -1.608475 1 -1.876276 1

N 0.941181 1 -2.387708 1 -0.439687 1

N -0.175836 1 0.965228 1 -2.448983 1

N -2.603697 1 -0.205162 1 0.441376 1

C -0.789356 1 -2.931243 1 -2.047202 1

C -0.123603 1 -3.338288 1 -0.745233 1

C 2.162398 1 -2.830659 1 -0.421179 1

C 3.386704 1 -2.062448 1 -0.306566 1

C 3.398094 1 -0.648310 1 -0.118766 1

C 4.662791 1 0.025875 1 -0.083762 1

C 5.800679 1 -0.735277 1 -0.223441 1

C 5.794291 1 -2.126460 1 -0.372878 1

C 4.587430 1 -2.777188 1 -0.412863 1

C 4.742024 1 1.538225 1 0.130459 1

C 3.907053 1 2.289153 1 -0.918969 1

C 4.228445 1 1.860637 1 1.543979 1

C 6.166793 1 2.061498 1 0.032174 1

C -1.508139 1 -0.923587 1 -3.195391 1

C -0.277652 1 -0.098249 1 -3.457400 1

C -0.168647 1 2.186596 1 -2.869644 1

C -0.136242 1 3.388485 1 -2.088494 1

C -0.090609 1 3.408223 1 -0.660310 1

C -0.086911 1 4.666860 1 0.025797 1

C -0.168191 1 5.822277 1 -0.743550 1

C -0.227462 1 5.797188 1 -2.134262 1

C -0.202549 1 4.606434 1 -2.800103 1

C 0.047463 1 4.734257 1 1.550303 1

C 1.377183 1 4.126526 1 1.973018 1

C 0.005083 1 6.158410 1 2.090916 1

C -1.089620 1 3.951976 1 2.232540 1

C -2.848441 1 -1.825143 1 -1.387446 1

C -3.405177 1 -0.575240 1 -0.741398 1

C -3.235033 1 0.021067 1 1.527040 1

C -2.665162 1 0.339649 1 2.824989 1

C -1.292250 1 0.169558 1 3.151142 1

C -0.893567 1 0.393848 1 4.510962 1

C -1.832334 1 0.810637 1 5.412101 1

C -3.183812 1 1.015790 1 5.088683 1

C -3.579426 1 0.748399 1 3.798192 1

C 0.579366 1 0.182827 1 4.929811 1

C 1.483568 1 1.146044 1 4.152963 1

C 0.802224 1 0.459796 1 6.425135 1

C 1.008483 1 -1.270872 1 4.687864 1

H -1.426080 1 -3.600449 1 -2.312281 1

H -0.118488 1 -2.879850 1 -2.730277 1

H 0.217498 1 -4.224612 1 -0.788946 1

H -0.789832 1 -3.320152 1 -0.031537 1

H 2.281257 1 -3.783897 1 -0.525801 1

H 6.643992 1 -0.267438 1 -0.227497 1

H 6.630141 1 -2.610097 1 -0.397492 1

H 4.594833 1 -3.726889 1 -0.539989 1

H 3.925271 1 3.242635 1 -0.760094 1

H 4.260047 1 2.154091 1 -1.814913 1

H 2.999523 1 2.008650 1 -0.923215 1

H 4.238736 1 2.806654 1 1.720501 1

H 3.327725 1 1.550806 1 1.673613 1

H 4.781363 1 1.435674 1 2.205633 1

H 6.677224 1 1.623790 1 0.632202 1

H 6.461448 1 1.901965 1 -0.878542 1

H 6.132749 1 3.000923 1 0.167976 1

H -1.619350 1 -1.585849 1 -3.877220 1

H -2.276536 1 -0.345816 1 -3.215875 1

H -0.279694 1 0.285551 1 -4.341810 1

H 0.526301 1 -0.656937 1 -3.409239 1

H -0.185008 1 2.308520 1 -3.835743 1

H -0.185237 1 6.680954 1 -0.313500 1

H -0.248284 1 6.633637 1 -2.624206 1

H -0.256217 1 4.615517 1 -3.797819 1

H 1.499152 1 4.155017 1 2.915733 1

H 1.416918 1 3.206545 1 1.689150 1

H 2.121549 1 4.582871 1 1.560178 1

H 0.726271 1 6.694009 1 1.718387 1

H -0.810056 1 6.576382 1 1.899193 1

H 0.141112 1 6.162130 1 3.052636 1

H -0.967222 1 3.971077 1 3.177656 1

H -1.917550 1 4.386888 1 2.026673 1

H -1.094119 1 3.077180 1 1.917516 1

H -3.411690 1 -2.125743 1 -2.084164 1

H -2.847701 1 -2.547795 1 -0.703343 1

H -4.319295 1 -0.727486 1 -0.462737 1

H -3.397887 1 0.119169 1 -1.380982 1

H -4.216331 1 -0.007449 1 1.457102 1

H -1.558438 1 0.988663 1 6.339447 1

H -3.853263 1 1.295117 1 5.748672 1

H -4.550264 1 0.810384 1 3.550711 1

H 2.442794 1 0.974767 1 4.351909 1

H 1.414704 1 0.997225 1 3.190431 1

H 1.325762 1 2.051055 1 4.325350 1

H 1.746494 1 0.349846 1 6.654806 1

H 0.580291 1 1.372820 1 6.640160 1

H 0.301795 1 -0.115123 1 6.977479 1

H 0.486121 1 -1.878544 1 5.217786 1

H 0.860526 1 -1.505600 1 3.758500 1

H 1.926105 1 -1.407468 1 4.882836 1

0

---------------------------------------- End of file **GACJIE.mop**-----------------------------------

---------------------------------------- Begin of file **GACJIE.arc**-----------------------------------

SUMMARY OF RM1 CALCULATION, Site No: 999

MOPAC2009 (Version: 11.03W )

Wed Jun 25 20:46:25 2014

Empirical Formula: C39 H51 N4 O3 Ce = 98 atoms

RM1 EXTERNAL=spk.inp PRECISE NOINTER XYZ BFGS T=10D GNORM=0.25 +

NOLOG GEO-OK SCFCRT=1.D-10 charge=0

NUMERO DE COORDENAÇÃO = 8

PETERS TEST WAS SATISFIED IN BFGS OPTIMIZATION

SCF FIELD WAS ACHIEVED

HEAT OF FORMATION = -119.53538 KCAL/MOL = -500.13602 KJ/MOL

TOTAL ENERGY = -7451.21465 EV

ELECTRONIC ENERGY = -92003.91578 EV

CORE-CORE REPULSION = 84552.70113 EV

GRADIENT NORM = 0.24213

DIPOLE = 10.23640 DEBYE POINT GROUP: C1

NO. OF FILLED LEVELS = 124

IONIZATION POTENTIAL = 7.956230 EV

HOMO LUMO ENERGIES (EV) = -7.956 -0.460

MOLECULAR WEIGHT = 763.973

COSMO AREA = 549.85 SQUARE ANGSTROMS

COSMO VOLUME = 852.04 CUBIC ANGSTROMS

MOLECULAR DIMENSIONS (Angstroms)

Atom Atom Distance

H 77 H 54 12.65987

H 72 H 88 12.13385

H 64 H 82 10.88841

SCF CALCULATIONS = 421

COMPUTATION TIME = 21 MINUTES AND 51.422 SECONDS

FINAL GEOMETRY OBTAINED CHARGE

RM1 EXTERNAL=spk.inp PRECISE NOINTER XYZ BFGS T=10D GNORM=0.25 +

NOLOG GEO-OK SCFCRT=1.D-10 charge=0

NUMERO DE COORDENAÇÃO = 8

Ce -0.01505675 +1 0.17478069 +1 -0.04091009 +1 0.2837

O 2.36862228 +1 -0.04648925 +1 0.05380716 +1 -0.3927

O -0.11735546 +1 2.56001049 +1 -0.24703194 +1 -0.3928

O -0.46930087 +1 0.01964185 +1 2.30675719 +1 -0.3930

N -1.47413020 +1 -1.42875465 +1 -1.83857126 +1 -0.2015

N 0.92216776 +1 -2.29645146 +1 -0.36976909 +1 -0.2895

N -0.14999802 +1 1.10603317 +1 -2.53238087 +1 -0.2893

N -2.56744319 +1 -0.21789861 +1 0.60974269 +1 -0.2896

C -0.80314686 +1 -2.78313557 +1 -2.05799617 +1 -0.0354

C -0.14803253 +1 -3.27905211 +1 -0.74786677 +1 -0.0211

C 2.12814092 +1 -2.83624554 +1 -0.27736447 +1 0.2268

C 3.35764860 +1 -2.14784054 +1 -0.00531861 +1 -0.3422

C 3.46084977 +1 -0.73978249 +1 0.12330994 +1 0.4003

C 4.74238601 +1 -0.11293527 +1 0.30644483 +1 -0.1917

C 5.83735992 +1 -0.92743936 +1 0.41487889 +1 0.0102

C 5.73851510 +1 -2.33481658 +1 0.32930582 +1 -0.2298

C 4.52987456 +1 -2.92965107 +1 0.11698791 +1 0.0237

C 4.84738569 +1 1.38896409 +1 0.37972167 +1 0.0950

C 4.26527543 +1 2.01028046 +1 -0.89358961 +1 -0.1865

C 4.09750550 +1 1.88821069 +1 1.61610653 +1 -0.1890

C 6.29868733 +1 1.87437262 +1 0.49507216 +1 -0.1935

C -1.60641817 +1 -0.76203683 +1 -3.20624567 +1 -0.0353

C -0.31689699 +1 0.01787515 +1 -3.55298124 +1 -0.0212

C -0.10367618 +1 2.32122902 +1 -3.05702562 +1 0.2267

C -0.01803905 +1 3.56196641 +1 -2.34091606 +1 -0.3421

C -0.06095336 +1 3.66120114 +1 -0.92735703 +1 0.4004

C -0.05837894 +1 4.94726272 +1 -0.28326203 +1 -0.1917

C 0.05237786 +1 6.05539795 +1 -1.07950499 +1 0.0102

C 0.13698469 +1 5.96395961 +1 -2.48736878 +1 -0.2298

C 0.09478808 +1 4.74793319 +1 -3.10307647 +1 0.0237

C -0.16686056 +1 5.04378288 +1 1.21706102 +1 0.0950

C 1.06308544 +1 4.39562959 +1 1.85514822 +1 -0.1891

C -0.23058278 +1 6.49504112 +1 1.71204548 +1 -0.1935

C -1.44908863 +1 4.35077463 +1 1.68790827 +1 -0.1865

C -2.88790197 +1 -1.70157195 +1 -1.32930592 +1 -0.0354

C -3.44172953 +1 -0.46414626 +1 -0.58553809 +1 -0.0213

C -3.22318403 +1 -0.24687538 +1 1.76007648 +1 0.2268

C -2.65861493 +1 -0.11501604 +1 3.07279483 +1 -0.3422

C -1.26747084 +1 -0.01467220 +1 3.32674700 +1 0.4005

C -0.77100308 +1 0.02877021 +1 4.67597023 +1 -0.1918

C -1.69011374 +1 0.03242526 +1 5.69061253 +1 0.0102

C -3.08071036 +1 -0.02795058 +1 5.44542753 +1 -0.2298

C -3.55274169 +1 -0.10715362 +1 4.16853123 +1 0.0237

C 0.71295153 +1 0.07595944 +1 4.93629055 +1 0.0949

C 1.28331988 +1 1.37857195 +1 4.37231432 +1 -0.1890

C 1.05114854 +1 0.03519092 +1 6.43267483 +1 -0.1935

C 1.38997074 +1 -1.13585371 +1 4.28881410 +1 -0.1864

H -1.51174706 +1 -3.55414321 +1 -2.44697373 +1 0.0675

H -0.01845648 +1 -2.70688259 +1 -2.84962744 +1 0.0940

H 0.20135896 +1 -4.33081196 +1 -0.90139709 +1 0.0684

H -0.87724016 +1 -3.35781968 +1 0.09722279 +1 0.1007

H 2.25989277 +1 -3.93292932 +1 -0.42954304 +1 0.0801

H 6.84603393 +1 -0.53282090 +1 0.57508321 +1 0.1038

H 6.64567347 +1 -2.93539040 +1 0.43283320 +1 0.1114

H 4.46793473 +1 -4.02111551 +1 0.04370753 +1 0.0870

H 4.48820768 +1 3.08572947 +1 -0.96538470 +1 0.0553

H 4.67488502 +1 1.54077541 +1 -1.79910354 +1 0.0544

H 3.16893833 +1 1.94000451 +1 -0.96183096 +1 0.0869

H 4.19523681 +1 2.97715318 +1 1.74184124 +1 0.0564

H 3.01506776 +1 1.68907877 +1 1.58469768 +1 0.0868

H 4.47557590 +1 1.42415565 +1 2.53848785 +1 0.0580

H 6.79977416 +1 1.50871328 +1 1.40274939 +1 0.0580

H 6.91080931 +1 1.58115050 +1 -0.36972204 +1 0.0576

H 6.35878274 +1 2.97231413 +1 0.54811646 +1 0.0617

H -1.84141415 +1 -1.49232650 +1 -4.01824628 +1 0.0675

H -2.47116566 +1 -0.05461184 +1 -3.21350860 +1 0.0942

H -0.38652267 +1 0.36474241 +1 -4.61441721 +1 0.0683

H 0.59646932 +1 -0.62836060 +1 -3.53974323 +1 0.1006

H -0.14299788 +1 2.45118242 +1 -4.16378121 +1 0.0802

H 0.08326592 +1 7.06988177 +1 -0.66879229 +1 0.1038

H 0.23342799 +1 6.88226867 +1 -3.07187755 +1 0.1114

H 0.15233359 +1 4.69101964 +1 -4.19576003 +1 0.0869

H 1.05416038 +1 4.49196887 +1 2.95141493 +1 0.0564

H 1.14637656 +1 3.31666141 +1 1.65247014 +1 0.0868

H 1.99741363 +1 4.85466802 +1 1.50093315 +1 0.0580

H 0.66761388 +1 7.07359476 +1 1.45273384 +1 0.0580

H -1.10439762 +1 7.03555052 +1 1.32116432 +1 0.0576

H -0.30880349 +1 6.54924996 +1 2.80874745 +1 0.0617

H -1.66133303 +1 4.55531618 +1 2.74834699 +1 0.0552

H -2.32621723 +1 4.68870541 +1 1.11821428 +1 0.0545

H -1.41666374 +1 3.25337982 +1 1.60861040 +1 0.0869

H -3.58765384 +1 -2.00096944 +1 -2.14692916 +1 0.0676

H -2.88996062 +1 -2.57446775 +1 -0.63192752 +1 0.0941

H -4.52298078 +1 -0.64074189 +1 -0.35854538 +1 0.0684

H -3.44660647 +1 0.45654337 +1 -1.22141690 +1 0.1007

H -4.32796143 +1 -0.39759535 +1 1.76693816 +1 0.0802

H -1.39749330 +1 0.08404751 +1 6.74439059 +1 0.1038

H -3.76832457 +1 -0.01344654 +1 6.29473781 +1 0.1114

H -4.63272752 +1 -0.16201816 +1 3.99190832 +1 0.0869

H 2.35538495 +1 1.48668175 +1 4.59608145 +1 0.0563

H 1.19904118 +1 1.45726114 +1 3.27726781 +1 0.0867

H 0.77869315 +1 2.26134246 +1 4.79092024 +1 0.0581

H 2.13755602 +1 0.07012958 +1 6.60699510 +1 0.0617

H 0.63728573 +1 0.88992520 +1 6.98663330 +1 0.0580

H 0.69817412 +1 -0.88524462 +1 6.91927735 +1 0.0576

H 0.88256813 +1 -2.07431938 +1 4.55324812 +1 0.0545

H 1.42893182 +1 -1.08938082 +1 3.18976166 +1 0.0869

H 2.43792225 +1 -1.24082945 +1 4.60841183 +1 0.0552

---------------------------------------- End of file **GACJIE.arc**-----------------------------------


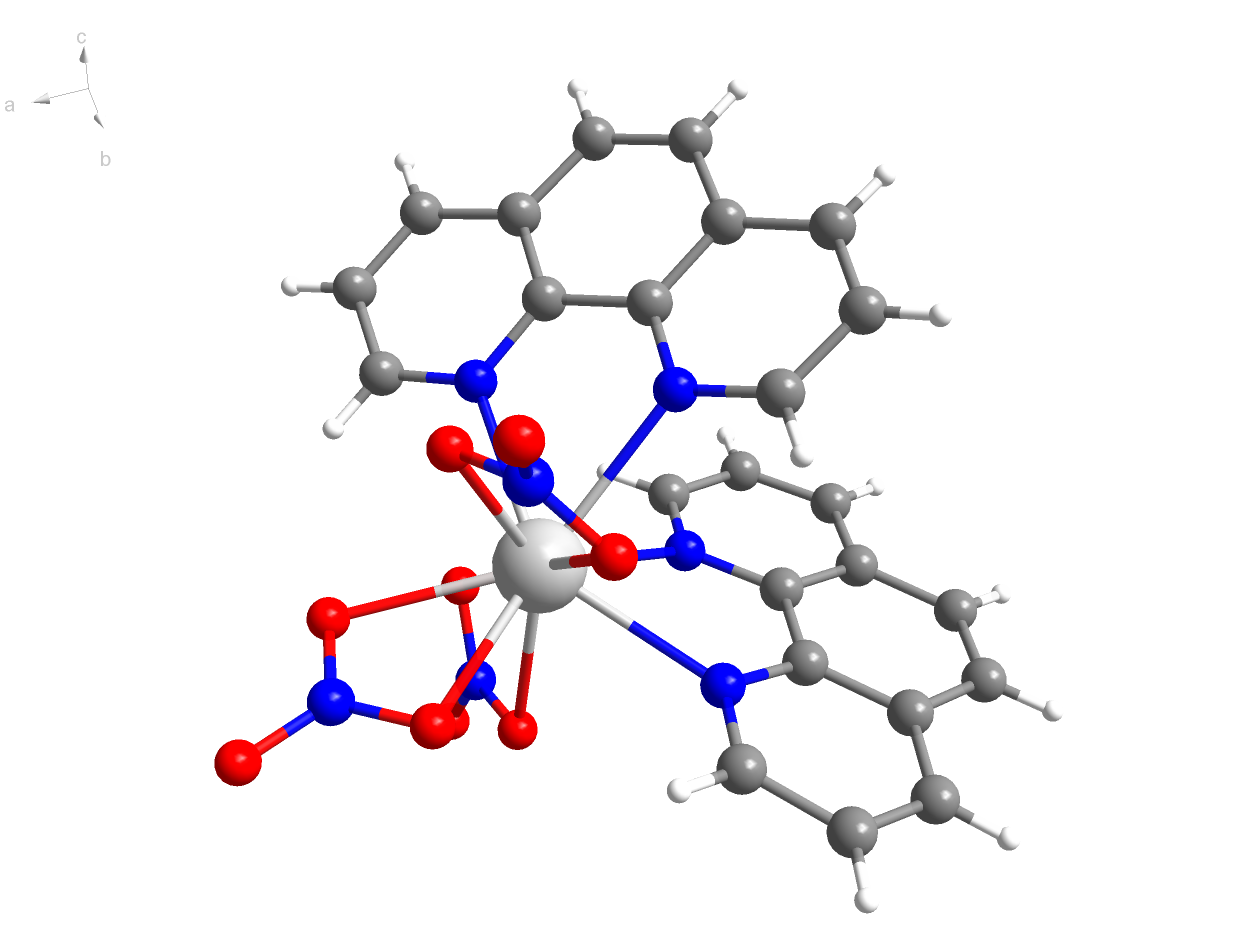


S3_Figura: Crystallographic structure of the prazeodimium complex (ECABAL).

---------------------------------------- Begin of file **ECABAL.mop**-----------------------------------

RM1 EXTERNAL=spk.inp PRECISE NOINTER XYZ BFGS T=10D GNORM=0.25 +

NOLOG GEO-OK SCFCRT=1.D-10

NUMERO DE COORDENAÇÃO = 10

Pr 0.000000 1 0.000000 1 0.000000 1

O 2.554400 1 0.000000 1 0.000000 1

O 1.648505 1 1.951758 1 0.000053 1

O 0.685038 1 0.407620 1 -2.428940 1

O 0.938338 1 -1.629789 1 -1.761045 1

N -1.984455 1 -0.985627 1 -1.456888 1

N -1.731584 1 1.680851 1 -0.973998 1

N 0.166040 1 -2.407547 1 0.973978 1

N -2.033739 1 -0.879335 1 1.456859 1

O 0.753092 1 0.260972 1 2.428977 1

O -0.638983 1 1.768657 1 1.761085 1

O 3.793295 1 1.760593 1 0.000000 1

O 1.491478 1 -1.078095 1 -3.778979 1

N 2.692125 1 1.249496 1 0.000000 1

N 1.038252 1 -0.783296 1 -2.696156 1

C -2.110961 1 -2.285466 1 -1.704786 1

C -3.289894 1 -2.852230 1 -2.179305 1

C -4.360890 1 -2.062451 1 -2.429913 1

C -4.247297 1 -0.670642 1 -2.235536 1

C -3.041166 1 -0.174370 1 -1.735843 1

C -5.304657 1 0.241713 1 -2.549847 1

C -5.147531 1 1.565774 1 -2.382595 1

C -3.936759 1 2.111487 1 -1.879692 1

C -0.917498 1 -3.008796 1 1.523117 1

C -3.712099 1 3.484926 1 -1.717603 1

C -2.543981 1 3.920614 1 -1.219895 1

C 1.274034 1 -3.127337 1 0.835549 1

N 0.071968 1 1.298560 1 2.696119 1

C -2.890229 1 1.241422 1 -1.523138 1

C -3.108093 1 -0.136959 1 1.705747 1

C -4.302427 1 -0.670565 1 2.179318 1

C -4.390578 1 -1.998350 1 2.429921 1

C -3.253895 1 -2.810894 1 2.235501 1

C -2.096270 1 -2.210075 1 1.735813 1

C 1.351771 1 -4.472951 1 1.219948 1

C 0.265054 1 -5.083862 1 1.718625 1

C -0.928923 1 -4.368861 1 1.880715 1

C -2.126871 1 -4.942223 1 2.383569 1

C -3.240004 1 -4.206587 1 2.550881 1

C -1.566091 1 2.991714 1 -0.835573 1

H -1.378776 1 -2.839327 1 -1.555068 1

H -3.341354 1 -3.769775 1 -2.323945 1

H -5.159626 1 -2.434103 1 -2.726334 1

H -6.110901 1 -0.087637 1 -2.875180 1

H -5.845846 1 2.139271 1 -2.601224 1

H -4.374435 1 4.094940 1 -1.955329 1

H -2.383398 1 4.833433 1 -1.130894 1

H 2.027697 1 -2.722709 1 0.470628 1

H -3.058906 1 0.780755 1 1.555079 1

H -5.036029 1 -0.118491 1 2.324916 1

H -5.189617 1 -2.369389 1 2.726295 1

H -4.011545 1 -4.610646 1 2.876154 1

H -2.140082 1 -5.844850 1 2.601256 1

H 0.303377 1 -5.983448 1 1.955348 1

H 2.152658 1 -4.939621 1 1.131939 1

H -0.770530 1 3.306084 1 -0.469651 1

O 0.139381 1 1.835004 1 3.778944 1

0 ---------------------------------------- End of file **ECABAL.mop**-----------------------------------

---------------------------------------- Begin of file **ECABAL.arc**-----------------------------------

SUMMARY OF RM1 CALCULATION, Site No: 999

MOPAC2009 (Version: 11.03W )

Tue Jan 21 17:51:33 2014

Empirical Formula: C24 H16 N7 O9 Pr = 57 atoms

RM1 EXTERNAL=spk.inp PRECISE NOINTER XYZ BFGS T=10D GNORM=0.25 +

NOLOG GEO-OK SCFCRT=1.D-10

NUMERO DE COORDENAÇÃO = 10

PETERS TEST WAS SATISFIED IN BFGS OPTIMIZATION

SCF FIELD WAS ACHIEVED

HEAT OF FORMATION = -159.94852 KCAL/MOL = -669.22462 KJ/MOL

TOTAL ENERGY = -7593.09535 EV

ELECTRONIC ENERGY = -70741.54458 EV

CORE-CORE REPULSION = 63148.44923 EV

GRADIENT NORM = 0.23105

DIPOLE = 20.26235 DEBYE POINT GROUP: C1

NO. OF FILLED LEVELS = 102

IONIZATION POTENTIAL = 10.380001 EV

HOMO LUMO ENERGIES (EV) = -10.380 -2.429

MOLECULAR WEIGHT = 687.340

COSMO AREA = 453.32 SQUARE ANGSTROMS

COSMO VOLUME = 603.49 CUBIC ANGSTROMS

MOLECULAR DIMENSIONS (Angstroms)

Atom Atom Distance

H 54 H 47 11.95435

H 43 O 12 10.33174

H 51 O 13 9.68100

SCF CALCULATIONS = 325

COMPUTATION TIME = 10 MINUTES AND 37.046 SECONDS

FINAL GEOMETRY OBTAINED CHARGE

RM1 EXTERNAL=spk.inp PRECISE NOINTER XYZ BFGS T=10D GNORM=0.25 +

NOLOG GEO-OK SCFCRT=1.D-10

NUMERO DE COORDENAÇÃO = 10

Pr -0.41666749 +1 -0.18501932 +1 -0.02277662 +1 -0.2953

O 2.20508364 +1 -0.13988673 +1 -0.00158356 +1 -0.3496

O 1.28404751 +1 1.81194565 +1 -0.05047483 +1 -0.3485

O 0.18145850 +1 0.31251778 +1 -2.56658061 +1 -0.3362

O 0.58055442 +1 -1.70248806 +1 -1.92618276 +1 -0.3884

N -2.34820640 +1 -1.03081061 +1 -1.55198841 +1 -0.1800

N -1.95772338 +1 1.63602138 +1 -0.91813842 +1 -0.1919

N 0.02315732 +1 -2.52118688 +1 0.89955966 +1 -0.1934

N -2.26870263 +1 -1.11799572 +1 1.56084217 +1 -0.1838

O 0.44238066 +1 0.05301488 +1 2.48502876 +1 -0.3448

O -0.99775251 +1 1.53857439 +1 1.89350449 +1 -0.3943

O 3.38375672 +1 1.62101021 +1 0.42523595 +1 -0.2092

O 1.44027385 +1 -0.97028583 +1 -3.76919076 +1 -0.2292

N 2.33727928 +1 1.11984337 +1 0.15455766 +1 0.5627

N 0.78442568 +1 -0.78151390 +1 -2.78933096 +1 0.5585

C -2.51368066 +1 -2.31023554 +1 -1.91712177 +1 0.1338

C -3.51717205 +1 -2.72771165 +1 -2.82824697 +1 -0.1811

C -4.35607718 +1 -1.79431374 +1 -3.38014031 +1 0.0043

C -4.19720811 +1 -0.43064183 +1 -3.03741783 +1 -0.0550

C -3.18773787 +1 -0.08477912 +1 -2.13121960 +1 0.1060

C -5.02145887 +1 0.59005247 +1 -3.60963924 +1 -0.0853

C -4.81701213 +1 1.89606915 +1 -3.31370177 +1 -0.0664

C -3.77468288 +1 2.28931774 +1 -2.41545987 +1 -0.0646

C -0.85910159 +1 -3.09653166 +1 1.80957124 +1 0.1075

C -3.50445522 +1 3.65168893 +1 -2.13131324 +1 0.0083

C -2.47763385 +1 3.97171667 +1 -1.28361097 +1 -0.1817

C 1.18005286 +1 -3.15974628 +1 0.65456728 +1 0.1791

N -0.19354336 +1 1.10812115 +1 2.78663214 +1 0.5569

C -2.97866585 +1 1.31535247 +1 -1.80784635 +1 0.1089

C -3.36165458 +1 -0.43364461 +1 1.92856614 +1 0.1344

C -4.31265353 +1 -0.93794489 +1 2.85223691 +1 -0.1809

C -4.11685984 +1 -2.17296223 +1 3.41387861 +1 0.0044

C -2.96502281 +1 -2.91769142 +1 3.06562037 +1 -0.0548

C -2.06692925 +1 -2.36430174 +1 2.14541169 +1 0.1033

C 1.49885843 +1 -4.40687372 +1 1.25897256 +1 -0.1819

C 0.61227662 +1 -4.98721902 +1 2.12625565 +1 0.0087

C -0.60548326 +1 -4.32521395 +1 2.42387454 +1 -0.0643

C -1.55860399 +1 -4.87184080 +1 3.34063978 +1 -0.0667

C -2.69324509 +1 -4.19754800 +1 3.64563117 +1 -0.0847

C -1.70885900 +1 2.93573088 +1 -0.68537420 +1 0.1799

H -1.80949868 +1 -3.04181967 +1 -1.47417459 +1 0.1667

H -3.59512976 +1 -3.78936497 +1 -3.08292721 +1 0.1477

H -5.13812164 +1 -2.08972414 +1 -4.08899208 +1 0.1316

H -5.81489740 +1 0.28878681 +1 -4.30224787 +1 0.1280

H -5.43964967 +1 2.67847152 +1 -3.76170790 +1 0.1281

H -4.11216782 +1 4.43400950 +1 -2.60035226 +1 0.1312

H -2.22514600 +1 5.01277306 +1 -1.05445318 +1 0.1548

H 1.89450572 +1 -2.67233628 +1 -0.04787800 +1 0.2052

H -3.48816892 +1 0.57059905 +1 1.47811533 +1 0.1682

H -5.18116538 +1 -0.32304858 +1 3.10882465 +1 0.1480

H -4.83379943 +1 -2.58426619 +1 4.13375040 +1 0.1318

H -3.42097362 +1 -4.61278132 +1 4.35138156 +1 0.1282

H -1.34380395 +1 -5.84577696 +1 3.79435030 +1 0.1283

H 0.83561115 +1 -5.95003309 +1 2.60000999 +1 0.1314

H 2.45861300 +1 -4.87721075 +1 1.01850728 +1 0.1551

H -0.86664606 +1 3.17824300 +1 0.00240564 +1 0.2042

O -0.08019724 +1 1.64869114 +1 3.84592823 +1 -0.2331

---------------------------------------- End of file **ECABAL.arc**-----------------------------------
